# Known and Novel Per/Polyfluoroalkyl Substances (PFASs) in Serum of Tucson (AZ) Firefighters

**DOI:** 10.1021/acs.est.6c05168

**Published:** 2026-07-21

**Authors:** Yanan Chen, N. L. Dilani Perera, Shawn C. Beitel, John J. Gulotta, Miriam Calkins, Jefferey L. Burgess, Carrie A. McDonough

**Affiliations:** † Department of Chemistry, 6612 Carnegie Mellon University, Pittsburgh, Pennsylvania 15213, United States; ‡ Mel and Enid Zuckerman College of Public Health, 8041 University of Arizona, Tucson, Arizona 85724, United States; § Tucson Fire Department, Tucson, Arizona 85701, United States; ∥ Nicholas School of the Environment, 3065 Duke University, Durham, North Carolina 27708, United States

**Keywords:** AFFF, biomonitoring, bis-FMeSI, blood, firefighting, flame retardant, fluorotelomer, high-resolution mass spectrometry, human exposure, nontarget analysis, PFAS, suspect screening, UPFOS

## Abstract

Per/polyfluoroalkyl substance (PFAS) exposure is a serious concern for firefighters, but the contribution of firefighting activities to overall PFAS body burden remains poorly understood. We hypothesized that overlooked novel PFASs may be elevated in firefighter serum after fire suppression activities. We analyzed paired “baseline” (annual checkup) and “postfire” (within 35 h of fire) serum from 33 members of the Tucson Fire Department using high-resolution mass spectrometry. We measured concentrations of 49 PFASs via targeted quantitation and detected 15 in >50% of samples. Concentrations of target analytes were not significantly different between baseline and postfire paired samples. However, branched PFOS isomers and linear PFHxS were significantly elevated compared to the general population. Suspect screening enabled the identification of 26 additional PFASs. Two tentatively identified phosphate-containing PFASs were significantly (*p* < 0.01) elevated postfire. These chemicals may be associated with exposure to fluorinated aryl phosphate flame retardants or their combustion transformation products during fire response. Several other novel PFASs were detected in baseline and postfire samples, including bis­(trifluoromethylsulfonyl)­imide (bis-FMeSI). The presence of novel PFASs in serum from firefighters highlights the need for more research into their occurrence in occupationally exposed groups and the general population, exposure sources, and toxicokinetics.

## Graphical abstract

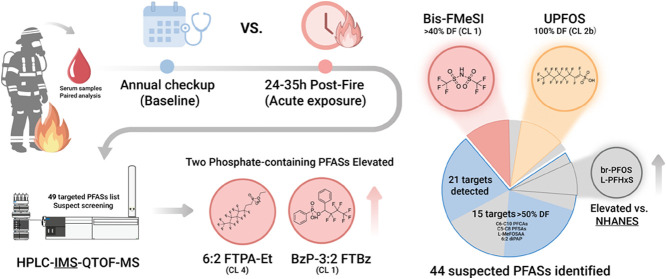

## Introduction

Hazardous chemical exposures contribute to elevated chronic disease risk among firefighters,^1–3^ and per/polyfluoroalkyl substance (PFAS) exposure is suspected to contribute to this risk.^4–7^ PFASs are associated with several adverse health outcomes, including cancer,^8–10^ but PFAS exposure sources and subsequent toxicokinetics remain poorly understood. In some studies, specific PFASs in blood from firefighters have been elevated compared with the general population,^4,11–13^ but few studies have screened these samples for novel PFASs not included on typical biomonitoring lists. The occurrence of novel overlooked PFASs may offer clues on sources contributing to PFAS exposure among firefighters and identify unique biomarkers of these occupational exposures.

Previous studies of PFAS exposure among firefighters primarily focused on cohorts with a history of using aqueous film-forming foams (AFFFs) containing long-chain (≥6 perfluorinated carbons) PFASs.^12^ These legacy PFASs (e.g., PFOS; PFOA) have high bioaccumulative potential and multisystem toxicity.^14,15^ Positive associations between years of firefighting service and elevated serum PFAS levels have been supported by several studies.^12,13,16^ Firefighters assigned to airport stations, who more frequently reported direct AFFF exposure, had higher concentrations of legacy PFASs (e.g., PFOS, PFNA) than those assigned to other stations.^4,11,17^ Notably, increases of up to 17% for PFHxS and 10% for PFNA have been observed following only three consecutive aircraft accident simulation training exercises.^17^ The long biological half-lives of these legacy PFASs lead to lasting body burdens^16,18^ and they may contribute to serum PFAS profiles for current firefighters along with a complex mixture of contemporary PFASs, including shorter-chain and ether-containing alternatives.

Use of historical AFFFs is limited now that health risks have been established, but many other potential sources of PFAS exposure remain, including PFAS-containing turnout gear,^19,20^ exposure to combustible materials (e.g., volatilized and particle-bound PFASs from building materials, furnishings, etc.),^21,22^ and modern short-chain fluorotelomer-based AFFFs.^23^ Elevated PFAS levels have also been reported in dust collected from fire station living areas,^24,25^ suggesting PFASs are transported back to the station on gear, potentially prolonging exposure past the initial event. A wide range of PFASs are used in commercial and industrial applications,^26^ so understanding current PFAS exposures among firefighters requires screening for PFASs beyond the well-studied perfluoroalkyl acids (PFAAs). In particular, PFASs with shorter biological half-lives may be underrepresented in traditional biomonitoring frameworks that rely on infrequent sampling.

Confident identification of novel PFASs in human blood is challenging due to trace PFAS levels and limited sample volume.^27^ As of 2024, at least 38 PFASs had been tentatively or unequivocally identified via high-resolution mass spectrometry (HRMS) in blood samples from human cohorts,^27^ including firefighters,^28^ communities with contaminated drinking water,^29,30^ and the general population.^31,32^ Since then, more recent studies have continued to contribute detections of novel PFASs in firefighter cohorts and other impacted populations. However, much more work is needed to understand how occupational PFAS exposure profiles differ among cohorts and over time.^33^

In this study, we analyzed paired “baseline” (annual checkup) and “postfire” (within 35 h of a fire response) serum samples from 33 municipal firefighters at the Tucson Fire Department (TFD) in Tucson, AZ. We used liquid chromatography and quadrupole-time-of-flight mass spectrometry (LC-QTOF-MS) to screen for known and novel PFASs, characterizing firefighters’ baseline serum PFAS profiles and evaluating whether suspected PFASs were elevated following a fire suppression activity. We hypothesized that overlooked novel PFASs may be elevated in serum after fire suppression activities. Our data offer a picture of PFAS exposure among municipal firefighters facing modern-day PFAS exposures and identify two novel PFASs potentially associated with fire suppression activities.

## Materials and Methods

### Sample Collection

The Cancer Prevention in the Fire Service protocol was approved by the University of Arizona Institutional Review Board, protocol #1509137073. All subjects provided written consent before participation in the study. Baseline serum samples were collected during annual physical examinations between November 2015 and June 2017. Between February and December 2015, an additional blood sample was collected from each firefighter upon their arrival on shift the day following a fire suppression activity (24–35 h later). Serum samples were collected by a qualified phlebotomist in 10 mL serum separator tubes with no additives, then centrifuged at 1000–1300*g*. Serum was aliquoted into cryovials and stored at −80 °C, then frozen on dry ice and shipped to Stony Brook University for analysis and later to Carnegie Mellon University (CMU) for further analysis.

Samples were collected following five events (duration and number of involved individuals in parentheses) including fires impacting a one-story house (18 min; *N* = 7), a school (33 min; *N* = 3), a car and house (21 min; *N* = 2), a business including exterior property (20 min; *N* = 9), and another large business (43 min; *N* = 12). Job duties completed during fire response were self-reported to have been pump operation (5), fire attack (18), salvage (9), rehab (3), rapid intervention crew (3), ventilation (4), secondary search (1), and fire cause investigation (1). Several firefighters performed multiple tasks. All study participants were male, aged 23–56 years. Time in the fire service ranged from 0.2 to 17 years (Table S1). Years of service were significantly correlated with age (*r* ^2^ = 0.49; *p* < 0.01).

### Sample Preparation and Data Acquisition

Paired serum samples (100 μL each) from 33 individuals (*N* = 66) were spiked with mass-labeled PFASs (Wellington Laboratories; Table S2) and prepared via Agilent Enhanced Matrix Removal (EMR) Lipid cartridge cleanup on a positive pressure manifold under ultrahigh purity nitrogen as previously described.^34^ Ten percent of samples were prepared and analyzed in duplicate. Triplicate LC-MS grade water (Fisher Scientific) and fetal bovine serum (Corning Life Sciences) samples were prepared alongside samples as solvent-based and matrix-matched blanks, respectively.

Serum extracts were screened on an Agilent 6545 QTOF-MS coupled to an Agilent Infinity 1290 LC system using analytical methods described in detail by Marciano et al.^34^ Extracts were injected (35 μL) onto an Agilent Poroshell C18 column with a binary mobile phase gradient of 100% Optima LC-MS grade methanol and 20 mM ammonium acetate in 100% Optima LC-MS grade water. Data were acquired using negative electrospray ionization (parameters in Table S3) with data-independent acquisition, fragmenting all ions with mass-to-charge (*m*/*z*) 65–1100 Da at three collision energies (0, 10, 35 eV) for targeted quantitation of 49 PFASs (Wellington Laboratories; Table S2) and simultaneous suspect screening. A calibration curve was analyzed at the beginning of the analytical run, and a continuing calibration verification (CCV) standard was analyzed after every 10 samples, as were analytical blanks and solvent blanks.

### Targeted Quantitative Analysis

All 49 analytes were quantified using 1/x-weighted linear calibration curves with linear quantitation ranges (listed in Table S2) limited to the range in which calibration points were measured within ±30% of expected concentrations, with >5 calibration points and *r* ^2^ > 0.99. Blank bovine serum (*N* = 3) fortified with targeted PFASs (2 ng/mL serum) was prepared alongside all samples to evaluate analyte recoveries. Solvent blanks were used for blank censoring and method reporting limit (MRL) calculations. Though bovine serum blanks were also analyzed, they were not used in blank censoring as, like most biological materials, they contained trace PFASs that were not representative of background contamination arising from sample handling and preparation. The MRL for each compound was defined as the low end of the calibration range, except in six cases when this value was exceeded by the average concentration in solvent-based preparatory blanks plus three standard deviations (Table S2). CCV (*N* = 8) recoveries were within ±25% of expected concentrations for all compounds. MRLs were <0.1 ng/mL for 47 analytes and <0.5 ng/mL for the remaining two. Values <MRL were replaced with MRL/sqrt(2) and values <DL (detection limit; defined as having signal: noise <3) were replaced with 0 when calculating central tendencies and describing distributions.

Triplicate injections of National Institute of Standards and Technology (NIST) nonfortified pooled freeze-dried human serum standard reference material (SRM 1957) were analyzed to test quantitation accuracy, and recoveries were within ±30% of published values for all fortified compounds except PFDA (65.6 ± 0.7%) (Table S4). We also identified eight out of 11 additional PFASs previously detected in NIST SRM 1957 by other studies,^35–37^ though concentrations differed markedly for three analytes, perhaps due to different sample preparation and analytical methods, including the use of different internal standards.

Five samples chosen at random were prepared and analyzed in duplicate, and relative percent differences (RPDs) were <30% in most instances (Table S5), though high variability was observed for 6:2 diPAP (average RPD 68.3 ± 59.2%; *N* = 4 pairs). In cases where branched isomers were evident in samples but were not present in the standards used for calibration, concentrations for summed branched isomers were estimated based on the response factor for the linear isomer. In cases where branched isomers were present in the analytical standard, both total isomers and linear isomers were measured, and branched isomers were determined by subtracting the linear from the total concentration.

### HRMS Suspect Screening

Suspect screening was performed in Agilent Profinder using an in-house database adapted from the NIST PFAS List v1.7.^38^ Screening filters were set as follows: exact mass match ±5.0 ppm, ion count ≥2, and peak height >10^3^ cps. For duplicate samples, hits were required to be detected in both samples, and areas were averaged. Peak abundances were normalized to the most appropriate mass-labeled analytical standard based on suspected structure and retention time (Table S6) to estimate relative abundance. Data were then blank-censored (remove all peaks with area ≤10× maximum peak area in blank serum) and filtered to remove peaks with RTs outside ±4 min of predictions based on the RT vs *m*/*z* relationship for PFAS standards to reduce false positives^39^ (Figure S1). Censored values were replaced with zero. Peaks with the same *m*/*z* (±5 ppm) and the same RT (±0.1 min) were considered to be the same chemical. In cases where chromatography suggested the presence of branched and linear isomers, the entire group of peaks was integrated, and the peak areas represent the summed isomers.

On the basis of initial suspect screening data, a subset of samples with high-abundance peaks of interest was prioritized for further analyses (method details in Table S3). Targeted MS/MS to isolate specific parent ions for fragmentation was performed using the same LC method hyphenated with an Agilent 6560 ion mobility spectrometer (IMS-QTOF-MS) using a narrow isolation width (∼1.3 Da) to minimize interference. The RT, MS1 spectrum, and MS2 fragmentation spectra were then compared to determine the identification confidence. In some cases, isomers were further differentiated by determining the collision cross section (CCS) via IMS. We acquired standards and confirmed tentative identifications where possible by analyzing at least one serum sample containing the tentatively identified PFAS of interest along with serum spiked with the analytical standard (Table S7). All peaks were assigned confidence levels (CLs) based on the PFAS confidence index,^40^ ranging from CL1 (confirmed with a standard) to CL4 (molecular formula with no additional structural information).

### Statistical Analysis

Neither PFAS concentrations nor internal standard-normalized peak abundances were normally distributed based on the Shapiro–Wilk test in R.^41^ Independent groups were compared via the Kruskal–Wallis rank sum test in R. Paired data were compared using a Wilcoxon signed-rank test, and significantly different groups were visualized using the package *ggstatsplot*.^42^ Linear correlations between variables were evaluated using the Pearson correlation coefficient, while monotonic associations were evaluated using the Spearman rank correlation coefficient. In all statistical tests, *p* < 0.01 was defined as significantly different from the null hypothesis, and only chemicals detected in at least 30% of samples were considered.

## Results and Discussion

Out of 49 targeted PFASs (listed in Table S2), 21 were detected in at least one sample, and 15 were above MRLs in ≥50% of samples (considered “frequently detected”). There were 44 PFASs tentatively identified via suspect screening (Table S6), including 18 that were included in the target list, providing complementary coverage of both well-characterized and poorly studied PFASs. All frequently detected target analytes were also identified via suspect screening, though for some low-level PFASs (e.g., MeFOSAA), detection frequencies from suspect screening were much lower. All other targets missed by suspect screening were detected infrequently and at low concentrations in targeted analysis.

### Targeted PFAS Analysis

Concentrations of analytes frequently detected above MRLs are summarized in Table 1. All concentration data are provided in Tables S8 and S9. These frequently detected target PFASs included PFCAs with six to 10 perfluorinated carbons (PFHpA to PFUdA) and PFSAs with five to eight perfluorinated carbons (PFPeS to PFOS), including summed branched isomers of PFHxS, PFHpS, and PFOS. Several shorter-chain PFAAs (e.g., PFBA, PFPeA, and PFPrS) had lower detection frequencies. Among precursors, L-MeFOSAA and 6:2 diPAP were frequently detected, while 6:2 FTS was detected in 15% of samples from both groups. Perfluoroalkyl sulfonamides (FASAs) and very long-chain PFAAs (PFHxDA; PFODA; PFDoS; PFTrDS) were not detected above MRLs in any serum samples. The absence of FASAs may be due to their propensity to partition to the red blood cell fraction^43,44^ as only serum was collected in this study.

**Table 1:** A Summary of Median Concentrations (ng mL^–1^ Serum; 95% Confidence Interval in Parentheses) for Frequently Detected (≥50% above MRL) PFASs in Postfire and Baseline Serum Samples Compared to NHANES PFAS Levels in Adult Males for the Nearest Collection Year^c^

	baseline (*N* = 33)		postfire (*N* = 33)		NHANES		
	DF (%)	median (95% CI)	DF (%)	median (95% CI)	sampling year	median (95% CI)	sample size
perfluoroalkyl carboxylates							
PFHpA	85	0.039 (0.013–0.140)	76	0.034 (0.013–0.095)	2013–2014	<0.1	1032
PFOA	100	1.75 (1.29–4.85)	100	1.95 (1.20–4.34)	2015–2016	1.80 (1.60–2.00)	964
PFNA	100	0.522 (0.285–0.878)	100	0.492 (0.281–0.807)	2015–2016	0.600 (0.600–0.700)	964
PFDA	100	0.206 (0.102–0.305)	100	0.176 (0.119–0.327)	2015–2016	0.100 (0.100–0.200)	964
PFUdA	100	0.074 (0.031–0.190)	100	0.073 (0.029–0.192)	2015–2016	<0.1	964
perfluoroalkyl sulfonates							
PFBS	58	0.035 (0.023–0.116)	55	0.033 (0.023–0.105)	2013–2014	<0.1	1032
PFPeS	91	0.098 (0.024–0.440)	91	0.116 (0.024–0.474)			
br-PFHxS^a^	100	0.491 (0.234–1.71)	100	0.552 (0.242–1.78)			
L-PFHxS	100	2.94 (1.52–10.8)	100	3.27 (1.53–11.5)	2015–2016	1.60 (1.50–1.70)	964
br-PFHpS^a^	100	0.055 (0.030–0.114)	100	0.062 (0.029–0.130)			
L-PFHpS	100	0.271 (0.151–0.604)	100	0.287 (0.148–0.579)	2017–2018	0.300 (0.200–0.300)	843
br-PFOS^b^	100	4.45 (2.07–10.4)	100	4.22 (1.97–9.96)	2015–2016	2.00 (1.80–2.20)	964
L-PFOS	100	3.94 (2.14–8.74)	100	3.66 (1.83–7.90)	2015–2016	4.00 (3.50–4.70)	964
pre-PFAAs							
L-MeFOSAA	58	0.034 (0.100–0.310)	73	0.041 (0–0.218)	2015–2016	<0.1	964
6:2 diPAP	91	0.181 (0.019–0.219)	88	0.160 (0–0.259)			

a Branched PFHxS and PFHpS concentrations estimated based on response factors for the linear isomers.

b br-PFOS concentration estimated based on response factor for the total linear and branched isomer mix in the analytical standard.

c NHANES DL = 0.1 ng mL^–1^ serum; DF = detection frequency (percentage of samples in which peaks were detected >MRL).

Several PFASs were frequently detected but had average levels <MRL, meaning that concentrations could not be measured confidently, though the presence of a peak was confirmed. In these cases, concentrations are expected to be low, as the MRL (which was <0.06 ng mL^–1^ for all relevant analytes) is considered an upper-bound value. Analytes in this category included several longer-chain PFCAs (PFDoA, PFTrDA, and PFTeDA) and PFSAs (PFNS and PFDS), FOSA, L-EtFOSAA, ADONA, and two chlorinated ether PFSAs frequently detected <MRL, Cl-PFENS and Cl-PFEUdS, which are emerging PFASs with increasing presence in human serum.^45^ Semiquantitative data for these analytes are discussed in the following sections on HRMS suspect screening. The remainder of this section focuses on target analytes that were >MRL in ≥50% of samples.

### Comparison of Paired Postfire and Baseline Samples

No target analytes were significantly elevated in postfire samples compared to baseline via the Wilcoxon signed-rank test (*p* > 0.01), suggesting that fire suppression activities undertaken by these 33 firefighters did not contribute significant increases in serum levels of these analytes. Similarly, Hoxie et al. observed no significant correlation between plasma PFAS and length of time spent in a fire.^46^ The finding that frequently detected target PFASs did not increase significantly over the course of one firefighting event is perhaps unsurprising, given their long biological half-lives in humans. Reported elimination half-lives of approximately two to five years for PFOA and three to six years for PFOS^47^ indicate slow clearance and suggest potential to accumulate over extended periods such that short-term, event-specific contributions may not be readily detectable. For example, the minimum detectable difference (MDD) for L-PFOS in our cohort (*n* = 33, α = 0.01, power = 0.8) by Wilcoxon signed-rank test with a Pitman asymptotic relative efficiency correction (ARE = 0.955) was 0.96 ng L-PFOS mL^–1^ serum. If PFOS concentrations increased by this level following one typical fire event, a firefighter participating in five such events per year would gain 2.7–4.8 ng PFOS mL^–1^ serum annually. Over a ten-year career, serum concentrations would reach approximately 26.5–48.0 ng PFOS mL^–1^ serum. This predicted career-long accumulation from acute events would escalate to levels far exceeding the median value (3.94 ng L-PFOS mL^–1^ serum) observed in this cohort. The large discrepancy between the statistical detection threshold and observed body burdens suggests that contemporary, per-event exposure to these targeted PFASs does not lead to detectable contributions to serum levels.

Among baseline samples, several clusters of structurally similar PFAA homologues covaried (i.e., an individual with elevated levels of one chemical was likely to have elevated levels of the others), exhibiting significant (*p* < 0.01) linear relationships, most notably among C4–C6 PFSAs, C7–C8 PFSAs, and C8–C10 PFCAs. Correlations among PFASs in human samples are frequently observed and are likely due to common exposure sources as well as similar toxicokinetics among similar structures.^29^

Strong correlations between paired baseline and postfire concentrations were observed for total serum PFASs (∑_49_PFAS_POST_ = 0.99 × ∑_49_PFAS_BASELINE_ + 0.067; *r* ^2^ = 0.92; *p* < 0.01) and for all frequently detected individual targets (*r* ^2^ > 0.80; *p* < 0.01) except 6:2 diPAP. This further supports the conclusion that target PFAS concentrations primarily reflect cumulative long-term exposure rather than acute contributions from isolated fire suppression activities. Two sample pairs (TF-165/WA-2094 and TF-148/WA-2505) exhibited increases for more than 10 analytes in postfire samples, resulting in an increase in ∑_49_PFAS of 47% and 39%, respectively. These increases may be due to specific exposures that occurred but were not widely observed across any one event, suggesting that impacts of specific job activities on PFAS exposure deserve further study.

In previous studies, PFOS and PFHxS levels were correlated with the number of years individuals spent in the fire service,^12^ supporting the interpretation that PFAS burdens in firefighters reflect cumulative, long-term occupational exposure. In our cohort, no significant relationships were observed between target PFAS concentrations and years in the fire service, possibly due to our small cohort size. In contrast, several PFASs were positively associated with age, including L-PFHpS, br-PFHpS, and br-PFOS (Spearman ρ = 0.55–0.70, *p* < 0.01), which has also been seen in previous studies of firefighters.^46^ These trends are consistent with long biological half-lives of many PFASs and tendency to accumulate over time in human serum,^47^ suggesting that legacy PFASs in serum from this cohort reflect cumulative long-term exposure that may be influenced by prior nonfirefighting occupations and/or nonoccupational background sources (e.g., diet, drinking water, and consumer products), particularly given that more than 50% of firefighters were over 40 years of age. The aforementioned correlation observed between age and years in the fire service also introduces a degree of multicollinearity, which complicates the isolation of occupational duration as a driver of serum concentrations.

### Comparison to NHANES Levels

Sixteen targeted PFASs overlapped with analytes included in past US National Health and Nutrition Examination Survey (NHANES) monitoring. Of these, 11 were frequently detected. Two analytes (br-PFOS and L-PFHxS) were elevated in this study cohort compared with adult males from the closest available NHANES sampling year. Concentrations of nine PFASs were similar to those of NHANES, though 95% confidence intervals were wider here for some analytes, including L-PFOS and PFOA (Table 1). The remaining five overlapping PFASs that were detected in less than 50% of our samples had median levels below the NHANES detection limit (DL_NHANES_ = 0.1 ng mL^–1^) in data from the closest available time range.

Elevated PFOS and PFHxS in firefighter cohorts are typically attributed to the use and handling of historical electrochemically fluorinated (ECF) AFFFs,^12^ which contain significant amounts of linear and branched PFSAs and sulfonamide-based precursors.^48,49^ Chlorinated PFOS (one fluorine replaced by a chlorine; many positional isomers possible) has also been reported as a potential marker of exposure to ECF AFFF.^18,28^ Here, Cl-PFOS was frequently detected but always <MRL in postfire and baseline samples. While the presence of these PFASs is often associated with AFFF exposure, this cohort was not known to be involved in handling historical AFFFs during their time with the Tucson Fire Department.

L-PFHxS was elevated (median 2.94 and 3.27 ng mL^–1^ in baseline and postfire groups) compared to NHANES (median 1.60 ng mL^–1^) and has been noted in several other studies on serum PFAS profiles of firefighters, often along with elevated total PFOS.^6,12,50,51^ While L-PFOS levels were similar to NHANES, summed branched isomers (br-PFOS) were considerably greater (median of 4.45 and 4.22 ng mL^–1^ in baseline and postfire group, respectively) than NHANES (median of 2.00 ng mL^–1^) and made up an average of 53 ± 7% total PFOS in all samples. Many studies do not distinguish between linear and branched PFOS isomers, despite disparities in their elimination half-lives,^18^ and elevated PFOS observed among firefighters in some studies may be enhanced by contributions from branched isomers. Similar to this study, Burgess et al. and Hoxie et al. explicitly distinguished between L-PFOS and br-PFOS isomers and found that while L-PFOS levels were not consistently elevated compared to NHANES, br-PFOS levels were significantly higher across all studied fire departments.^46,51,52^

br-PFOS was the most abundant target analyte measured for 36% of all serum samples, whereas L-PFOS was most abundant in 27% of samples, and L-PFHxS was most abundant in the remaining 36%. In serum from most populations, L-PFOS is the most abundant targeted PFAS, with summed branched isomers making up a smaller portion of total PFOS than that seen here.^53,54^ However, branched isomers typically contribute a significant portion of PFSAs in serum from firefighter cohorts. br-PFOS contributed an average of 55% of total PFOS in serum collected from Australian firefighters (*N* = 120) in 2013–2014, and the br-PFOS contribution increased to 65% in 2018–2019, suggesting longer half-lives for branched isomers.^18^ The increase in %br-PFOS over time was driven in large part by 1m-PFOS (perfluoro-1-methylheptanesulfonate), which is estimated to have a biological half-life almost 3x longer than L-PFOS (11.5 years compared to 4 years).^18^ In contrast, branched isomers of PFSAs and FASAs are often found to be excreted via urine more readily than linear isomers in animal studies.^48^ Branched PFOS isomers can also be formed *in vivo* as products of branched sulfonamide-based precursors.^49,55^ The interplay of these processes likely influences temporal trends and half-lives for PFSAs in human populations, even after exposure has ceased.

Along with elevated levels of br-PFOS, branched isomers of PFHxS, PFHpS, and PFNS were detected in most samples (Figure S2). Based on estimated concentrations using linear isomer response factors, br-PFHpS made up 17 ± 8% of total PFHpS, and br-PFHxS made up 14 ± 2% of total PFHxS. br-PFNS was typically <MRL (as was L-PFNS), but chromatography suggests significant contributions from branched isomers (Figure S2). Few studies have reported on the occurrence or concentration of branched PFSAs besides PFOS,^56^ though they have been measured in serum from individuals with AFFF-impacted drinking water.^29,57^ The presence of branched PFSAs indicates exposure to ECF-based PFASs, a process which typically generates PFOS mixtures consisting of about 70:30 L- to br-PFOS.^58^ In a previous study of serum from residents of a community with AFFF-impacted drinking water, serum from males in the most impacted water district exhibited br-PFHpS contributing 19 ± 4% of total PFHpS and br-PFOS contributing 46 ± 7% of total PFOS.^29^ br-PFHxS was <DL in all serum samples but was detected in 100% of raw drinking water samples with a median concentration of 69 ng L^–1^ (about 24% total PFHxS) in the most impacted water district. In a population of mothers in Sweden, the average percent contribution of branched isomers to total PFSA burden was 6% and 33% for PFHxS and PFOS, respectively, with elevated total PFHxS and %br-PFHxS in serum from mothers living in areas with PFAS-impacted drinking water.^57^ The primary source of exposure to PFASs produced via ECF among our cohort is unknown.

### HRMS Suspect Screening Results

There were 44 suspected PFASs identified, including 18 that were already included on the target analyte list. Among the additional chemicals identified, four were confirmed with analytical standards (CL1), and one, six, and 17 were identified at CL2 (probable identification based on MS/MS), CL3 (candidate based on subclass-consistent elution and/or MS/MS), and CL4 (unequivocal molecular formula—no homologues or fragmentation spectra available), respectively. All annotations, SMILES, and CLs are presented in Table S6. Spectral and chromatographic lines of evidence for CLs 1–3 are also detailed further in the Supporting Information.

### Novel PFASs Elevated in Postfire Serum

Two phosphate-containing PFASs [BzP-3:2 FTBz (CAS 496032–46–3)—CL1; 6:2 FTPA-Et (CAS 61726–44–1)—CL4] exhibited significantly greater normalized abundance in paired postfire samples versus baseline samples via the Wilcoxon signed-rank test (Figure 1A). No features were significantly elevated in the baseline group compared to postfire samples. Abundances of these two features covaried (Figure S4) along with two tentatively identified (CL4) imidazole-based PFASs (PFPr-BzImid—C_10_H_5_F_7_N_2_ and PFPr-Benz-Imid-One—C_10_H_5_F_7_N_2_O). Both BzP-3:2 FTBz and 6:2 FTPA-Et were detected more frequently in the postfire (DF = 48%) than in the baseline group (DF = 18–21%).

**Figure 1:**
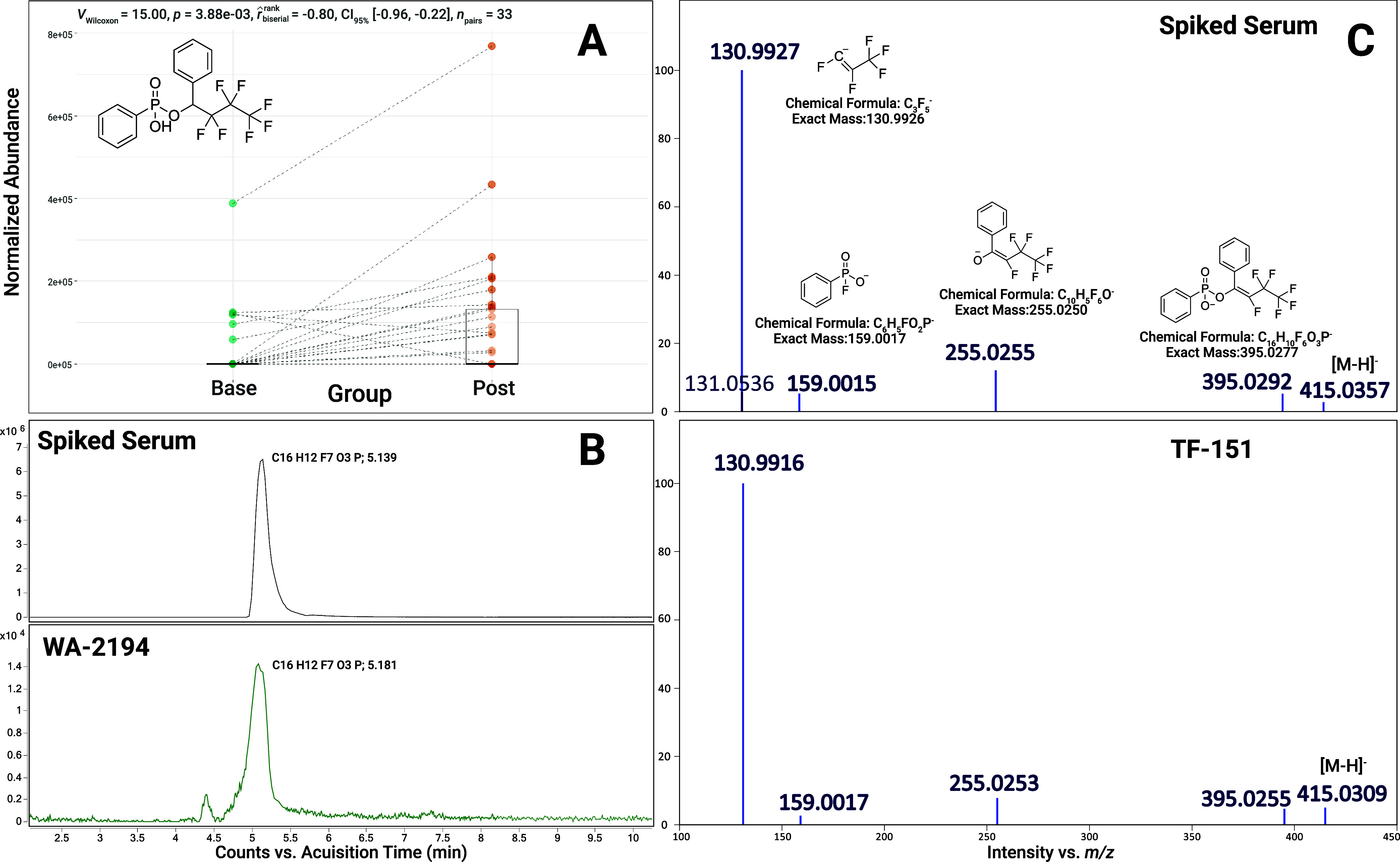
Identification of elevated BzP-3:2 FTBz (CL1) in postfire sample group. (A) Boxplots comparing normalized abundance in paired baseline and postfire samples for BzP-3:2 FTBz, which showed a significant difference (*p* < 0.01) in normalized abundance between sample groups. (B) Extracted ion chromatograms for spiked bovine serum (top) and a representative serum sample (WA-2194). (C) Fragmentation spectra from targeted MS/MS for standard-spiked calf serum (top, with tentative identification of major fragments) and a representative serum sample (TF-151; collision energy −20 eV). Due to sample volume limitations, injections for a clean TIC and MS/MS could not be done for the same sample. However, the MS/MS for WA-2194 with a wider isolation width is shown in Figure S3.

The identification of BzP-3:2 FTBz was confirmed in comparison to a purchased analytical standard (ChemBridge Corporation), with matching RT within 0.1 min, matching MS1 spectra, and four matching MS/MS fragments (Figure 1B,C). Due to sample volume limitations, we were not able to inject the same serum sample multiple times to acquire both a satisfactory chromatogram and a clean MS/MS spectrum, as obtaining these required using different isolation widths (narrow for clean fragmentation and wider for sufficient ion transmission for optimal peak shape) in these complex samples. For this reason, evidence obtained from analyzing the same peak in two different samples is presented. The MS/MS spectrum for sample WA-2194 is compared to that of fortified serum in Figure S3. All fragments seen in the standard were observed, although several other fragments were also seen in the serum sample due to the wider isolation width. Potential sources of exposure to this PFAS or other possible isomers are unknown; BzP-3:2 FTBz is included in several patents for therapeutics to inhibit phosphate transport, but no records of its widespread use in commercially available pharmaceuticals or other products were available. The structure of BzP-3:2 FTBz bears a resemblance to fluoroalkyl-substituted aryl phosphine oxides used in epoxies and polymers for flame retardancy,^59^ raising the possibility that this may be a transformation product (formed during combustion and/or via biological transformation) of a substance from this class of flame retardants. While the phosphate-containing and imidazole-containing PFASs covaried, detection did not appear to be consistently associated with specific fire response events. In 12 out of the 15 sample pairs in which BzP-3:2 FTBz was elevated, 6:2 FTPA-Et was also elevated. These included sample pairs from individuals who responded to fires at multiple businesses, a one-story house, and a car/house fire. Detection frequency and relative abundance of the imidazole-containing compounds were not significantly different among baseline and postfire groups.

### Bis-FMeSI in Serum

Bis­(trifluoromethylsulfonyl)­imide (bis-FMeSI; [C_2_F_6_NO_4_S_2_]^−^) was detected in 45% of baseline and 48% of postfire samples, with no significant difference in relative abundance among baseline and postfire groups. To confirm identification, a bis-FMeSI analytical standard spiked into bovine serum was analyzed with a subset of the serum samples. The standard matched the detected peak chromatographically and spectrally (Figure 2), confirming a CL1 identification. On the basis of normalized peak abundances compared to the analytical standard, we estimate that concentrations were fairly low (<0.15 ng mL^–1^) in all samples.

**Figure 2:**
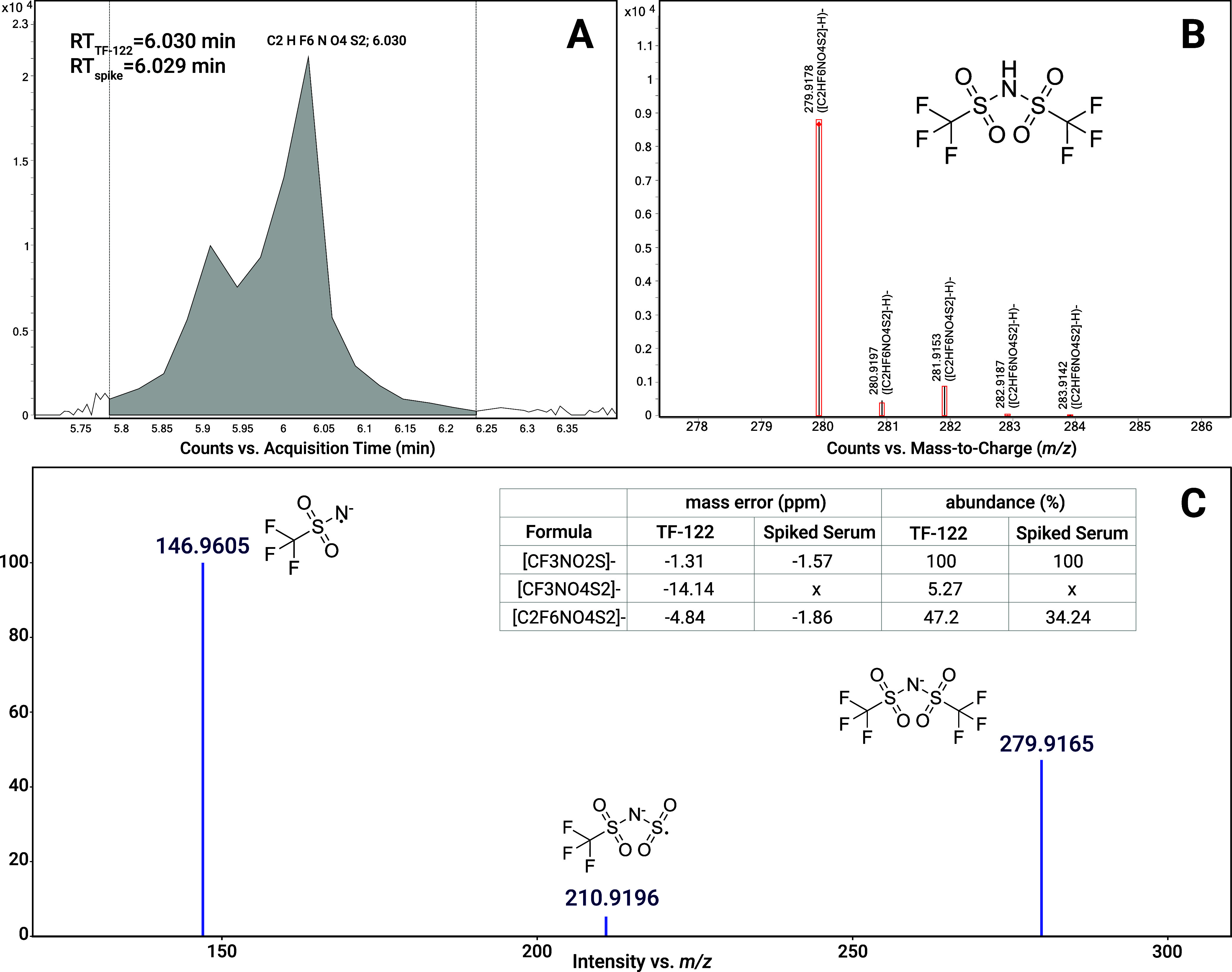
Bis-FMeSI in serum samples (shown here for serum sample TF-122) confirmed (CL1) via (A) retention time match, (B) MS1 match, and (C) MS2 spectrum, which matched one fragment found in the standard [CF3NO2S]- and another low-level fragment reported in PubChem spectra, [CF3NO4S2]-.

To our knowledge, this is the first confirmed identification of bis-FMeSI in human blood. A series of bis­(perfluoroalkylsulfonyl)­imides (bis-FASIs), including bis-FMeSI, were tentatively identified in one previous study of human serum from fluorochemical workers in China.^60^ Bis-FMeSI is used as an electrolyte in lithium-ion batteries (LiBs) and as an antistatic agent in poly­(vinylidene fluoride) (PVDF) composites in LiBs.^61^ It is widespread in the environment, with recent detections in dust from e-waste sites and surrounding regions^62^ and spatial trends suggesting atmospheric deposition.^61^ Bis-FMeSI has also been identified with varying confidence levels in drinking water studies.^63,64^

Bis-FMeSI has toxic effects on zebrafish at environmentally relevant levels and has exhibited hepatotoxicity in dosed mice.^61,65^ In a food web study, bis-FMeSI did not appear to be bioaccumulative in aquatic wildlife;^66^ however, it appeared to be similarly bioaccumulative to PFOA in liver tissue of dosed mice.^65^ While our sample size is too small to draw strong conclusions about exposure source or toxicokinetics, our data highlight knowledge gaps with respect to human bis-FMeSI exposure, bioaccumulation potential, and associated toxicity. Detection of bis-FMeSI in serum may suggest ongoing exposure, but further research on dominant bioaccumulation mechanisms, binding affinity for blood components, and biological half-lives is needed to understand how bis-FMeSI exposures impact the body burden for firefighters and other populations.

### Substituted PFSAs in Serum

Several substituted PFSAs [ketone-PFOS (k-PFOS), chlorinated PFSAs (Cl-PFSAs), hydrogen-PFOS (H-PFOS), and unsaturated PFOS (UPFOS)] were tentatively identified in serum. k-PFOS was present in both postfire and baseline samples with high detection frequency (≥70%). A series of C3, C6, and C8 chlorinated PFSAs (Cl-PFSAs) were tentatively identified in >30% of samples with a consistent *m*/*z* vs RT relationship indicative of a homologous series. Hydrogen-substituted PFOS (H-PFOS) was tentatively identified (CL3a) in one baseline/postfire sample pair with appreciable signal (1 × 10^4^ cps). UPFOS, k-PFOS, and other substituted PFSAs have previously been tentatively identified in serum from individuals exposed to AFFFs via contaminated drinking water^29^ and firefighting activity.^28^ UPFOS and H-PFOS were among the most elevated novel PFASs in serum from AFFF-dosed mice after a 10-day depuration, suggesting bioaccumulative potential similar to or greater than unsubstituted PFSAs.^49^ The source of substituted PFSA exposure among this cohort of firefighters (for whom appreciable AFFF usage for training and emergency response was not reported) is unknown. These chemicals may be associated with historical AFFF use, which is further supported by a significant correlation between the relative abundance of UPFOS and k-PFOS and firefighter age (Spearman ρ = 0.60–0.67, *p* < 0.01), but high detection frequencies suggest that direct AFFF use is not the only exposure source.

Tentatively identified UPFOS (CL2b) was widespread in serum (DF = 100%). UPFOS has previously been tentatively identified in electrochemically fluorinated AFFF and in mice dosed with AFFF mixtures.^49^ However, to our knowledge, the compound has not been unequivocally identified to date due to the lack of available analytical standards. UPFOS may be mistaken for the isomer perfluoroethylcyclohexanesulfonate (PFEtCHxS) in some scenarios, as MS2 fragmentation is similar at low collision energies.^40^ Here, UPFOS was distinguished from PFEtCHxS based on MS2 fragments unique to UPFOS^29^ and slightly different retention times when compared to an analytical standard for PFEtCHxS (Figure 3A,B). The identification was further corroborated by collision cross section (CCS) values obtained from the drift spectrum for [C8F15O3S]- via IMS (Figure 3C), which suggested the presence of two structural isomers in serum samples (CCS_UPFOS_ = 159.7 Å^2^, CCS_PFEtCHxS_ = 155.4 Å^2^), with CCS_PFEtCHxS_ matching the PFEtCHxS analytical standard (CCS = 155.5 Å^2^, deviation <0.1%). Chromatograms and drift spectra suggest minor contributions from PFEtCHxS, but the more extended structure with fragmentation consistent with UPFOS appears to be the dominant isomer.

**Figure 3:**
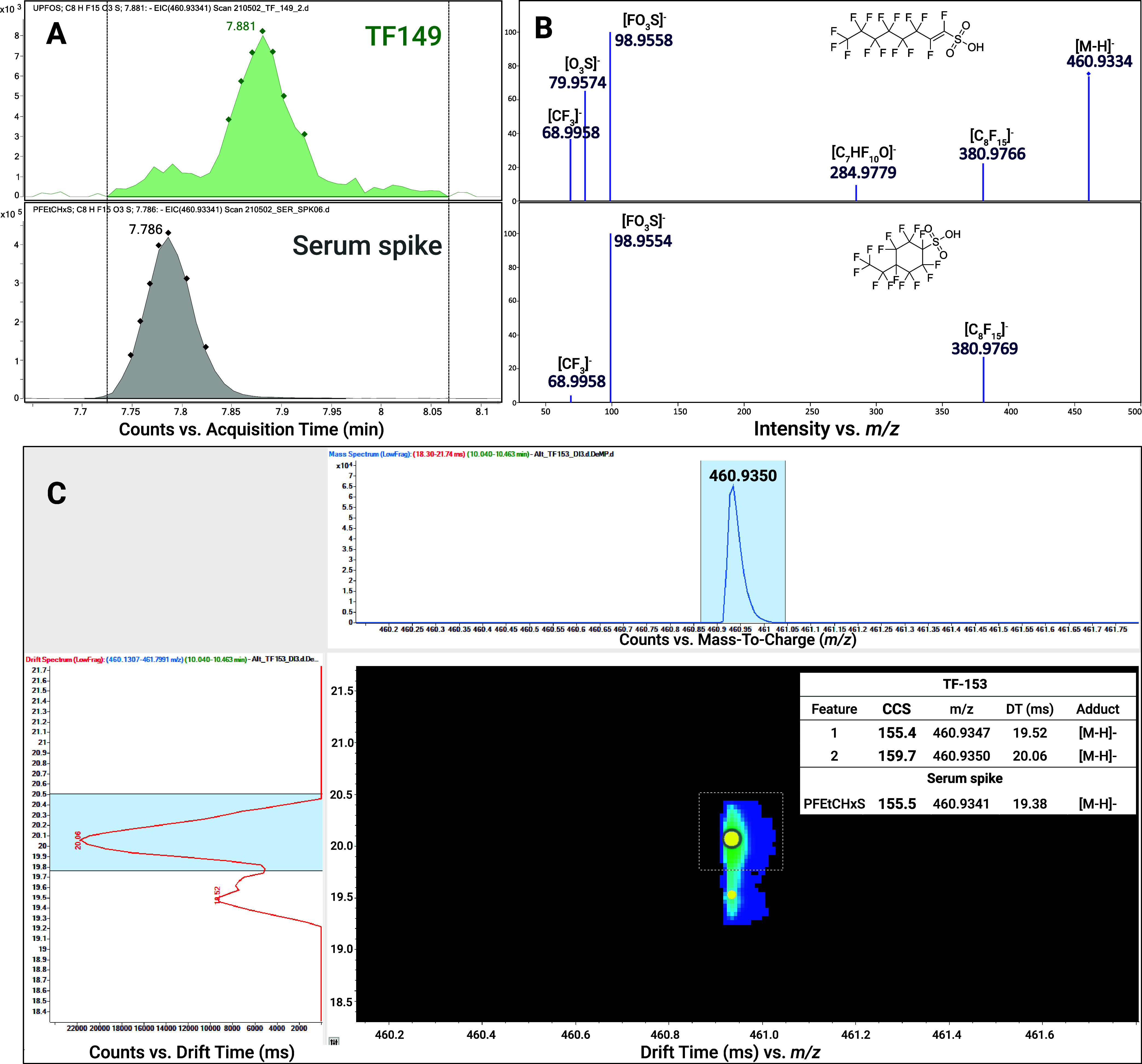
(A) EICs and (B) MS2 spectra at collision energy −40 eV for *m*/*z* = [C8F15O3S]- in a representative firefighter serum sample (TF-149) compared to bovine serum spiked with PFEtCHxS analytical standard. (C) The drift time versus *m*/*z* plot for [C8F15O3S]- in representative sample TF-153 and calculated CCS values compared to the PFEtCHxS analytical standard.

### Other Novel PFASs Detected in Serum

Beyond the compounds discussed in detail here, four perfluoroethercarboxylates (PFECAs) were identified with varying confidence, as were several structurally diverse PFAS precursors. Many ether-containing PFASs are being used at increasing volumes as replacements for older chemistries, and the presence of these chemicals may result from their applications in commercial products. Identification of PFE3DA (perfluoro-3,6,9-trioxadecanoic acid) was confirmed with an analytical standard (CL1; details in the SI), while OH-PFEBA and PFEPeA were both tentatively identified (CL4). PFEPeA appeared to be a distinct isomer of perfluoro-4-oxapentanoic acid (PF4OPeA) or a fragment of an unknown parent ion formed in-source, as the RT for the unknown peak was nearly 1 min later than the PF4OPeA analytical standard with the same exact mass. UPFE2OA was assigned CL3b based on supportive fragmentation (SI).

A sulfonamide-based precursor (FHxSAA; CL1) was also detected in >20% of baseline and postfire samples. Several poorly studied polyfluorinated precursors with potential to transform into PFAAs were also tentatively identified, two with CL3b (OH-3:2 FTS; 8:2 FTOBzA) and the others with CL4. While many fluorotelomers have well-studied degradation pathways leading to eventual formation of PFCAs, the transformation of the specific fluorotelomers listed here has not been well described. FHxSAA is expected to form FHxSA and, to some extent, PFHxS, *in vivo*. Exposure sources and biological transformation of several other tentatively identified potential PFAS precursors, including the benzimidazole-containing PFASs, require further study.

### Trends in Novel PFAS Occurrence

Correlation matrices of normalized abundances for tentatively identified PFASs with DF ≥30% in each sample group (Figure S4) were generally consistent with results from targeted analysis but revealed covariation of several other novel PFASs. Long-chain PFAAs tended to covary, with the strongest correlations among C6–C8 PFSAs, C8–C10 PFCAs, UPFOS, k-PFOS, and chlorinated PFSAs. As mentioned above, a group of four tentatively identified phosphate- and benzimidazole-containing PFASs was significantly correlated with each other, particularly in the postfire group. This clustering may indicate common sources or related transformation pathways linked to fluorinated additives or precursors associated with firefighting materials or combustion-derived mixtures, relationships that require further investigation.

This preliminary exploration of the modern firefighter PFAS exposome during baseline and postfire conditions demonstrates the value of pairing targeted biomonitoring with HRMS suspect screening for occupationally exposed populations. Targeted methods captured persistent legacy PFASs, whereas suspect screening revealed overlooked PFASs that may be more closely linked to recent or source-specific exposures. This study also raises further questions relevant to firefighters as well as the general population. We highlight several PFASs with poorly understood exposure sources and toxicokinetics that are detected frequently in serum from firefighters, underlining the importance of including HRMS analyses in studies of human PFAS exposure, particularly for groups with a high risk of occupational exposure. Information on which fire response was attended by which individual, years in the fire service, and age for each individual was available but did not appear to be significantly associated with serum PFAS profiles, likely due to the small sample size. Future studies with larger firefighter cohorts, repeated sampling, detailed activity records, and confirmation of tentatively identified analytes are needed to link specific job tasks and exposure scenarios to PFAS profiles and evaluate the utility of these candidate biomarkers across firefighter populations. Overall, PFAS profiles likely reflect combined contributions from occupational firefighting activities and background exposure sources, consistent with patterns observed for several targeted PFASs, as discussed above.

## Supplementary Material





## Notes

Tables summarizing firefighter metadata, tentatively identified PFASs, data acquisition parameters, and list of target analytes (XLSX)
PFAS relative abundances, representative branched/linear chromatography, correlation matrices, and summary of suspect screening lines of evidence (PDF)
