# Supplementary material for: Known and Novel Per/Polyfluoroalkyl Substances (PFASs) in Serum of Tucson (AZ) Firefighters
Source: Environ Sci Technol. 2026 Jul 21;60(30):20801–12. doi: 10.1021/acs.est.6c05168 (PMC13449899; doi:10.1021/acs.est.6c05168)

SUPPORTING INFORMATION

Known and Novel Per/Polyfluoroalkyl Substances (PFASs) in Serum of Tucson (AZ) Firefighters

Chen, Y.;[1] Perera, N. L. D.;[1] Beitel, S. C.;[2] Gulotta, J. J.;[3] Calkins, M.;[4] Burgess, J. L.;[2] McDonough, C. A.[1]*

1. Department of Chemistry, Carnegie Mellon University, Pittsburgh, PA, USA 15213
2. Mel and Enid Zuckerman College of Public Health, University of Arizona, Tucson, AZ, USA 85724
3. Tucson Fire Department, Tucson, AZ, USA 85701
4. Nicholas School of the Environment, Duke University, Durham, NC USA 27708

* Corresponding author: Carrie A. McDonough, cmcdonou@andrew.cmu.edu

Table of Contents

Figure S1. Retention time-mass relationship for filtered features....S2

Figure S2. Branched/linear chromatography of PFSAs S3

Figure S3. MS/MS spectra collected for the peak at 5.181 minutes in sample WA-2194 and in blank serum fortified with the BzP-3:2 FTBz analytical standard....S4

Figure S4. Frequently detected PFAS correlation matrix S5

Summary of suspect screening lines of evidence S6

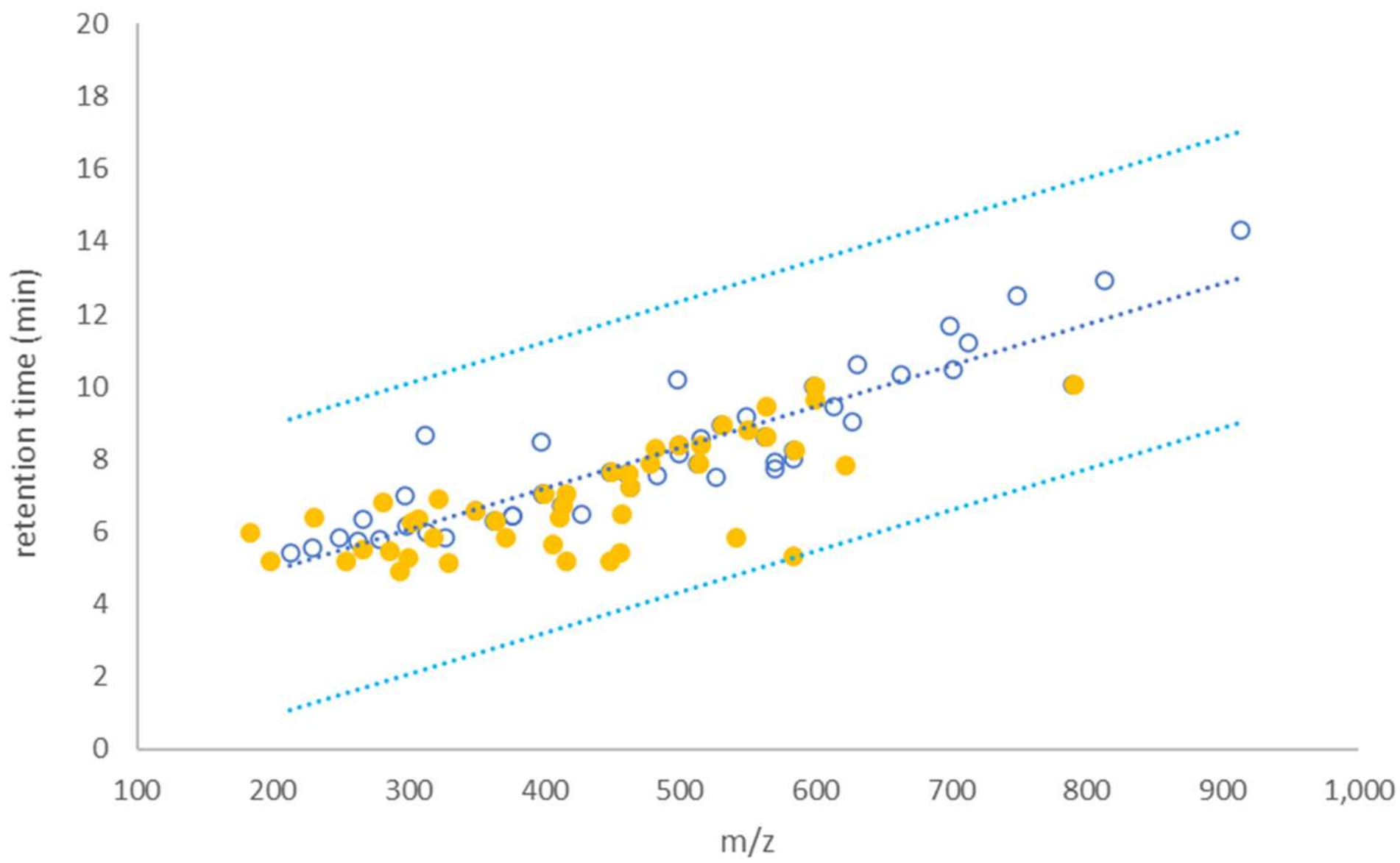

Figure S1. Retention time-mass relationship for filtered features. Features present in samples post-filtering (orange circles) and target analytes (blue open circles); dotted lines mark the range of RTs considered acceptable for suspected PFASs (±4 min of predicted RT based on target analyte m/z vs. RT relationship).

Figure S2. Branched/linear chromatography of PFSAs. Representative branched/linear chromatography evident for perfluoroalkyl sulfonates (PFSAs). All were integrated as summed isomers during suspect screening but summed branched isomers were quantified separately from the linear isomer during targeted quantitation.

PFHxS (C6)

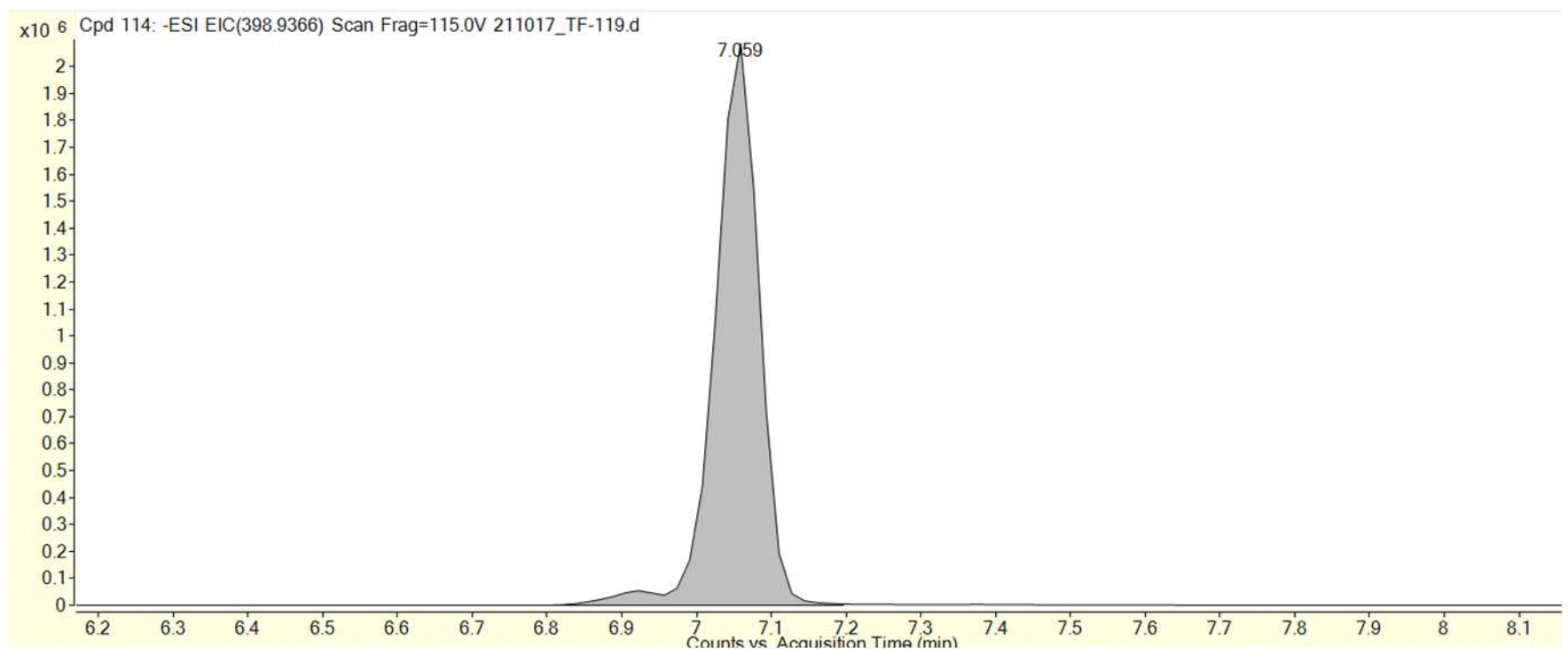

PFHpS (C7)

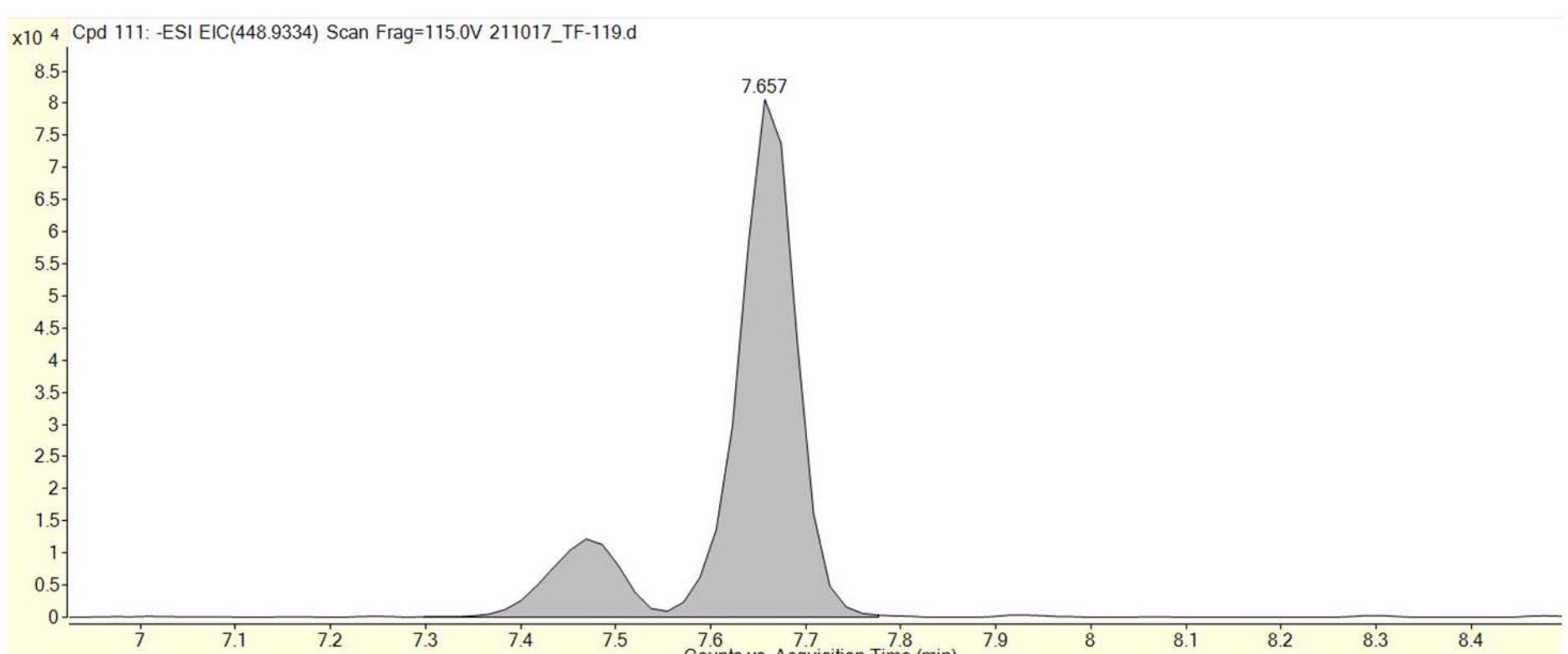

PFOS (C8)

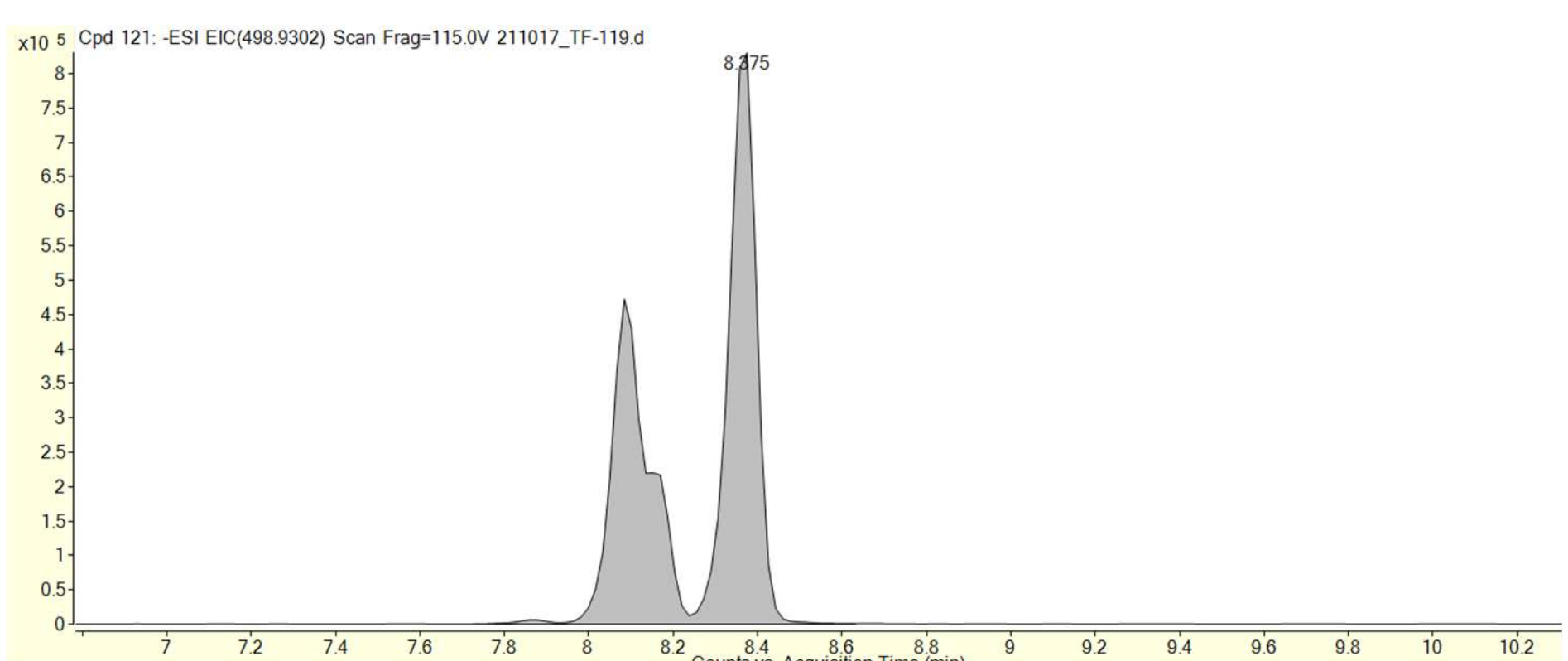

PFDS (C10)

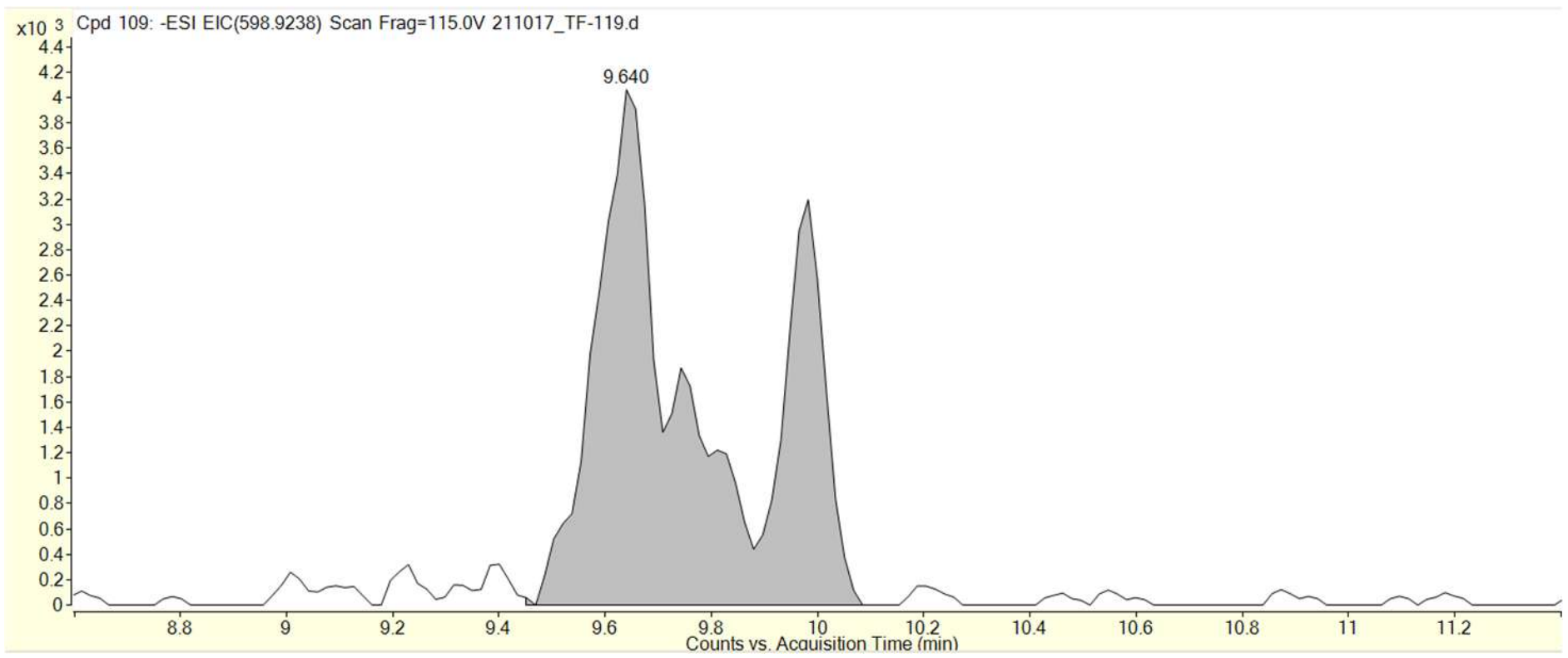

Figure S3. MS/MS spectra collected for the peak at 5.181 minutes in sample WA-2194 and in blank serum fortified with the BzP-3:2 FTBz analytical standard. Collected with collision energy 10 eV and medium (4 Da) isolation width.

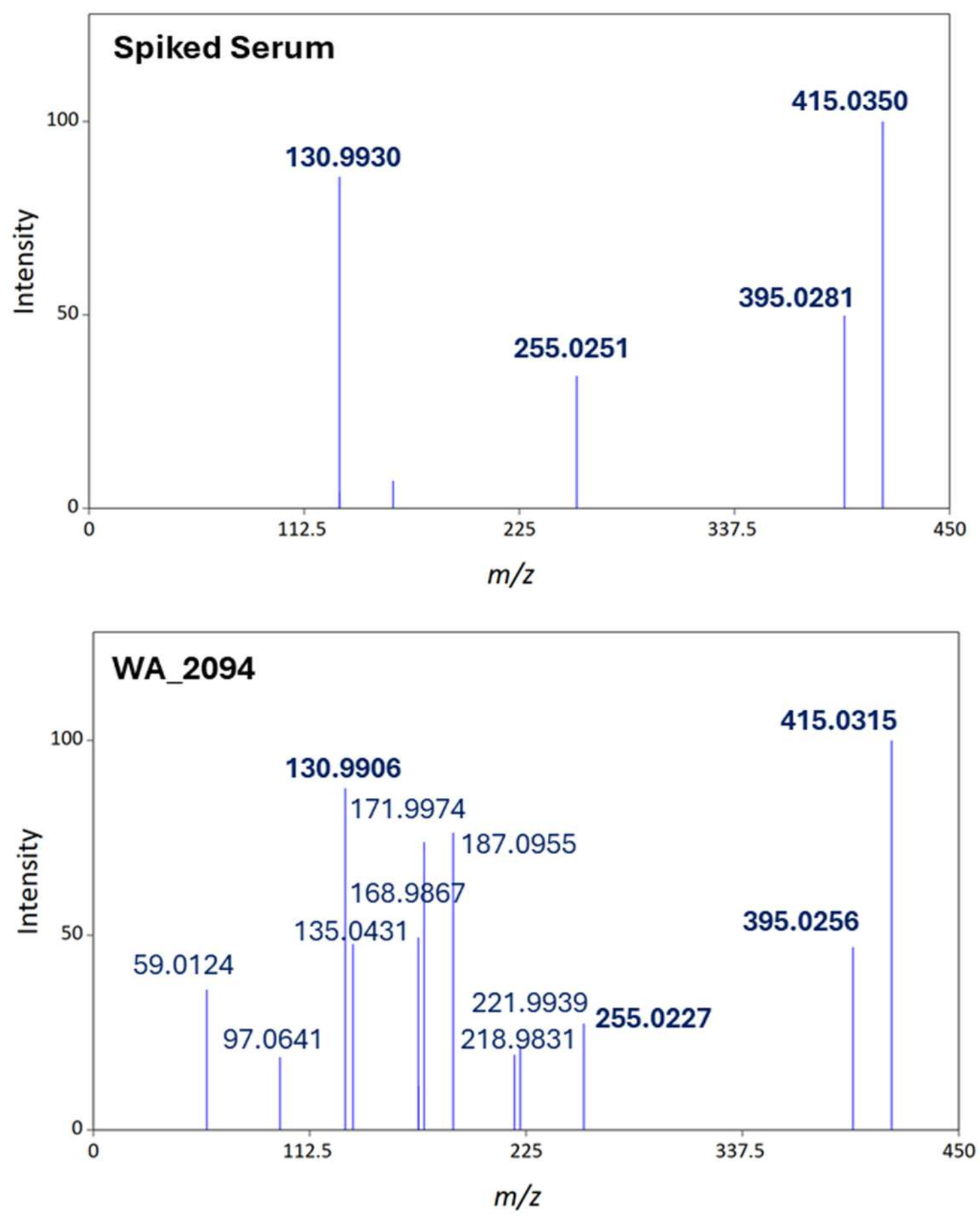

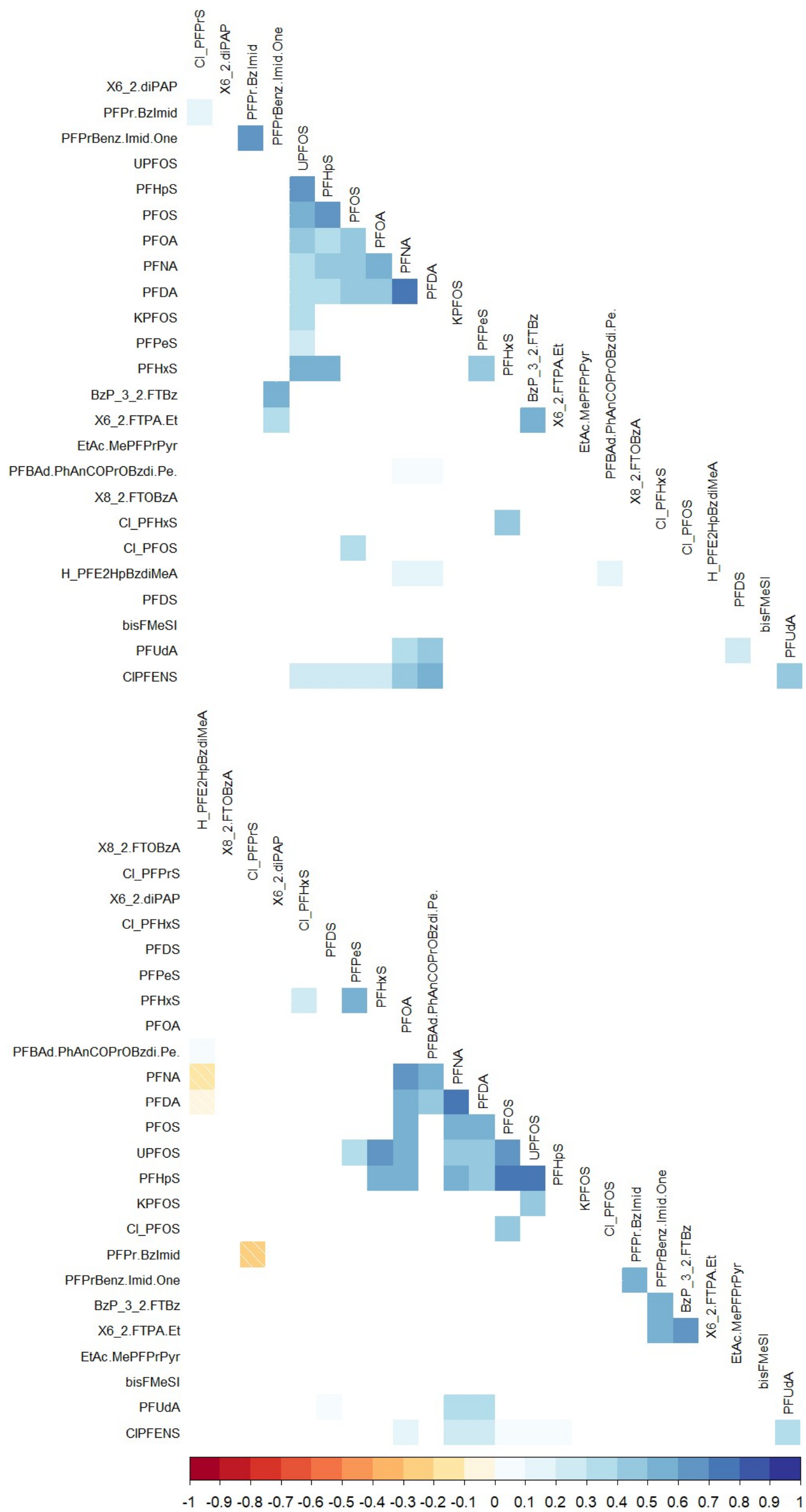

Figure S4. Correlation matrix for PFASs frequently detected via suspect screening. Correlation matrices for baseline (top) and post-fire (bottom) sample groups displaying associations between normalized peak abundances for all tentatively identified or unequivocally identified PFASs with DF $\geq$ 30%. Compounds are ordered based on hierarchical clustering. Colors correspond to r^2 value. Only correlations with p<0.01 are shown. The matrix was built using R package corrplot.[32,41]

SUSPECT SCREENING LINES OF EVIDENCE

CONFIDENCE LEVEL 1: For CL 1 identifications, representative extracted ion chromatograms (EICs), MS1, and MS2 spectra are compared to those for the analytical standard.

PFE3DA ($C_7HF_{15}O_5$)

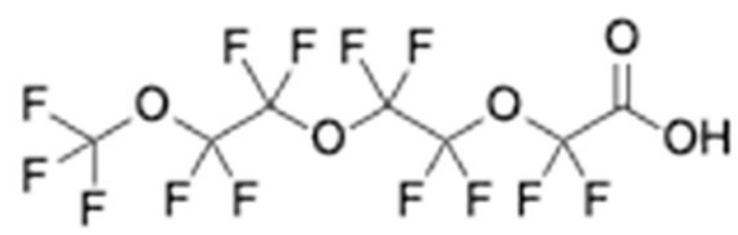

EIC **MS1**

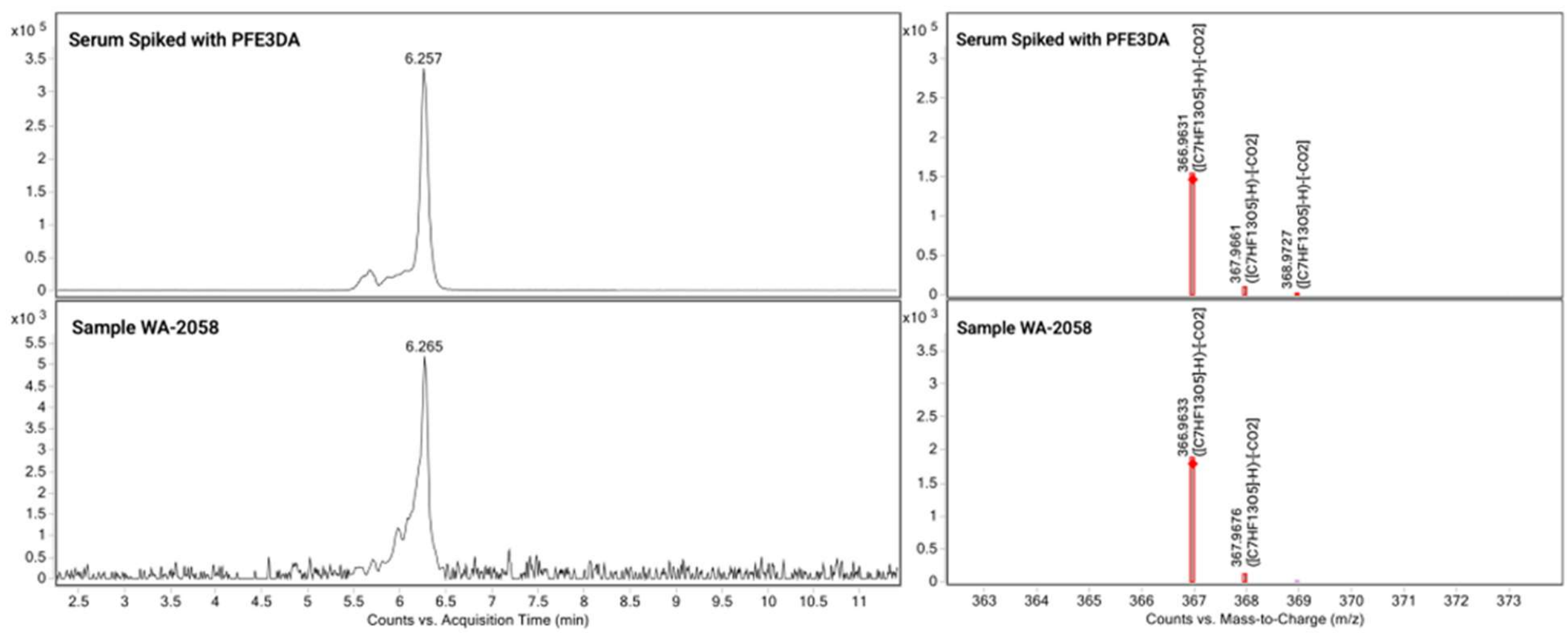

MS2 (from Data-Dependent Analysis; CE = - 40 eV)

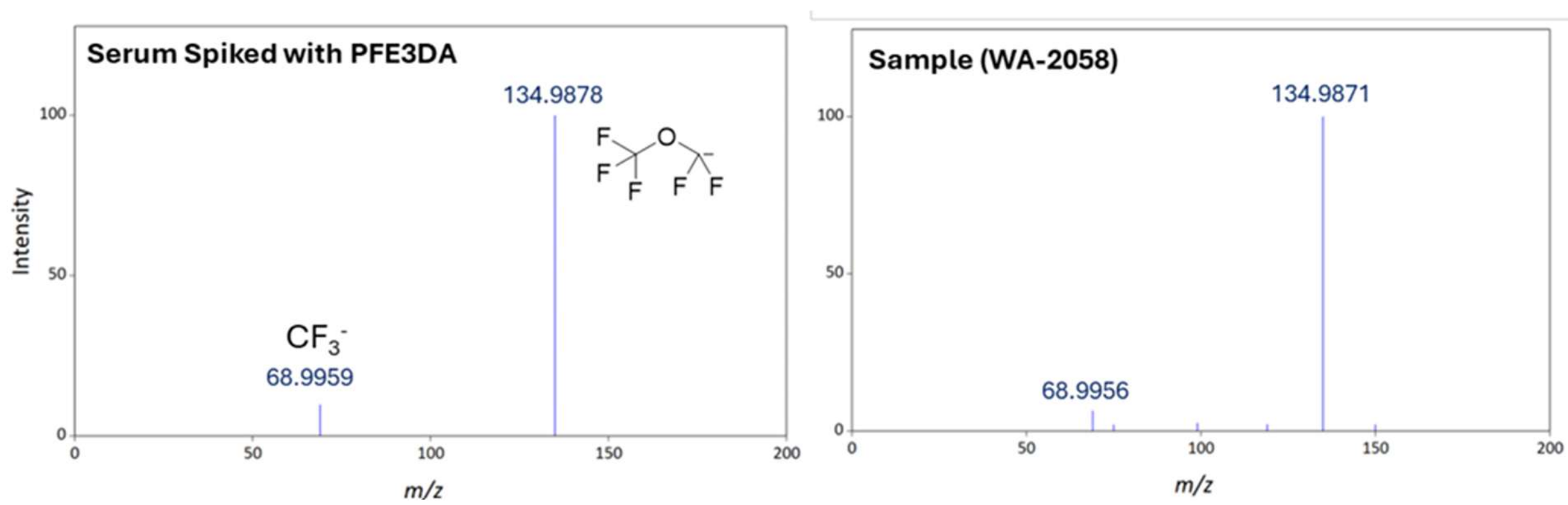

m/z	formula	mass error (ppm)		abundance (%)	
		Spiked Serum	WA-2058	Spiked Serum	WA-2058
68.9958	[CF3]-	1.63	-2.50	9.73	6.48
134.9875	[C2F5O]-	2.35	-2.76	100	100

MS2 (from Data-Dependent Analysis; CE = - 5 eV)

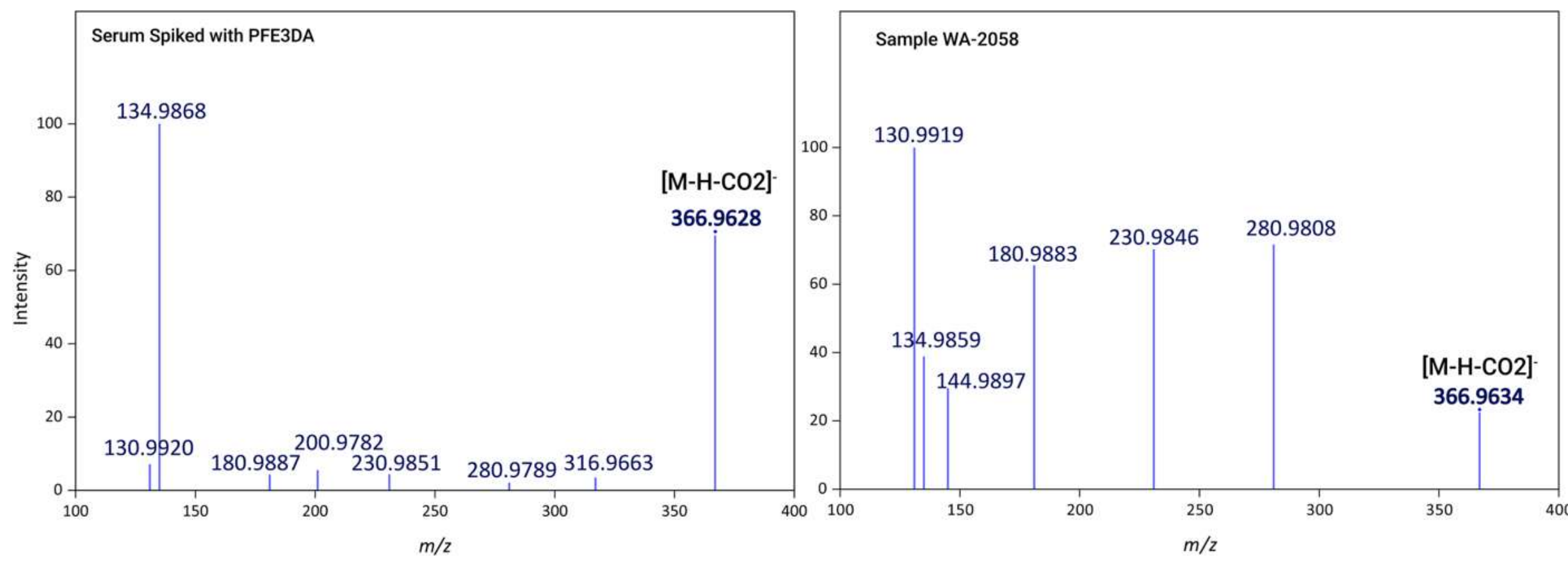

m/z	formula	mass error (ppm)		abundance (%)	
		Spiked Serum	WA-2058	Spiked Serum	WA-2058
130.9926	[C3F5]-	-4.13	-5.31	7.15	100
134.9875	[C2F5O]-	-5.3	-11.47	100	38.85
144.9907	[C6F3O]-	NA	-6.39	NA	29.56
180.9894	[C4F7]-	-3.57	-6.01	4.35	65.56
200.9792	[C3F7O2]-	-5.15	NA	5.55	NA
230.9862	[C5F9]-	-4.64	-6.82	4.40	70.19
316.9677	[C5F11O3]-	-4.41	NA	3.49	NA
366.9645	[C6F13O3]-	-4.65	-3.11	69.56	22.47

FHxSAA ($C_8HF_{13}NO_4S$)

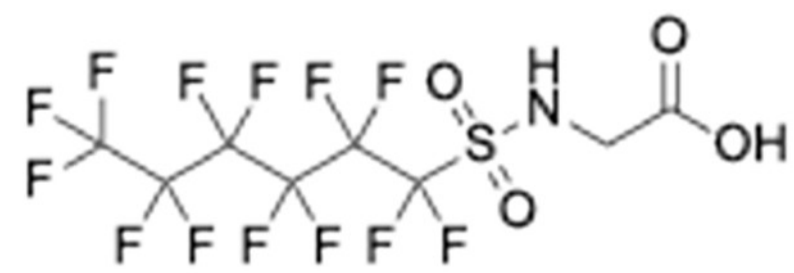

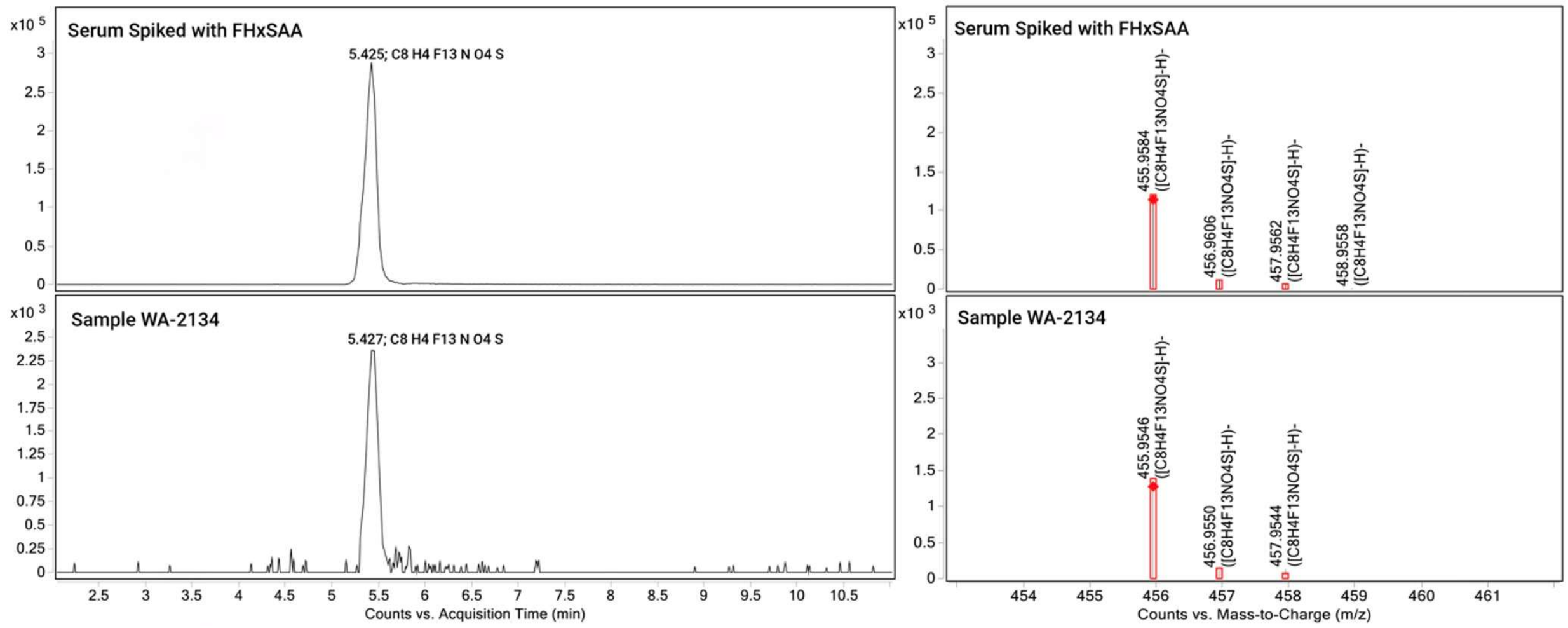

MS2 (from Data-Dependent Analysis; CE = - 20 eV)

Fragmentation spectra exhibited distinct relative abundance patterns for spiked serum compared to representative samples. This may be due to differences in the isomer mixture composition, as the analytical standard used was a mixture of linear and branched isomers.

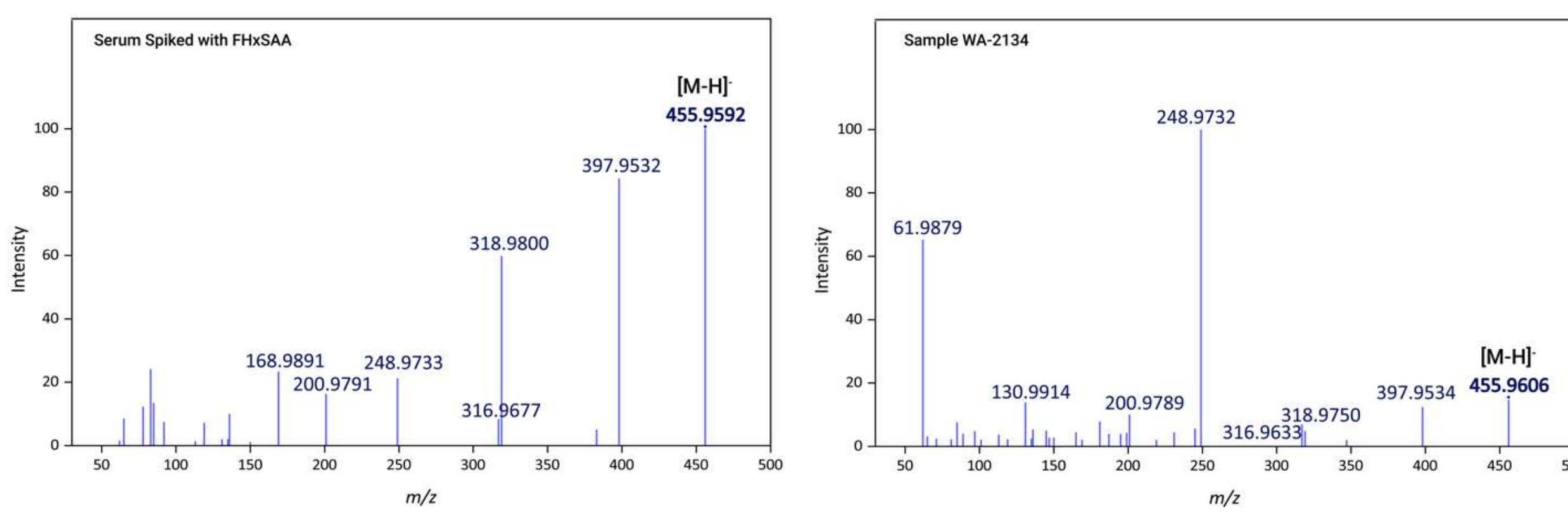

CONFIDENCE LEVEL 3a-3b: Four compounds were assigned level 3a-3b confidence. For these identifications, representative extracted ion chromatograms (EICs), MS1, and MS2 spectra with putative fragment annotations are shown.

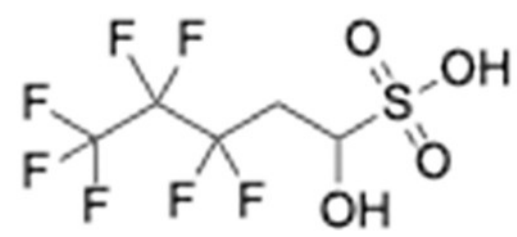

OH-3:2 FTS ($C_5H_5F_7O_4S$) – CL3b

EIC

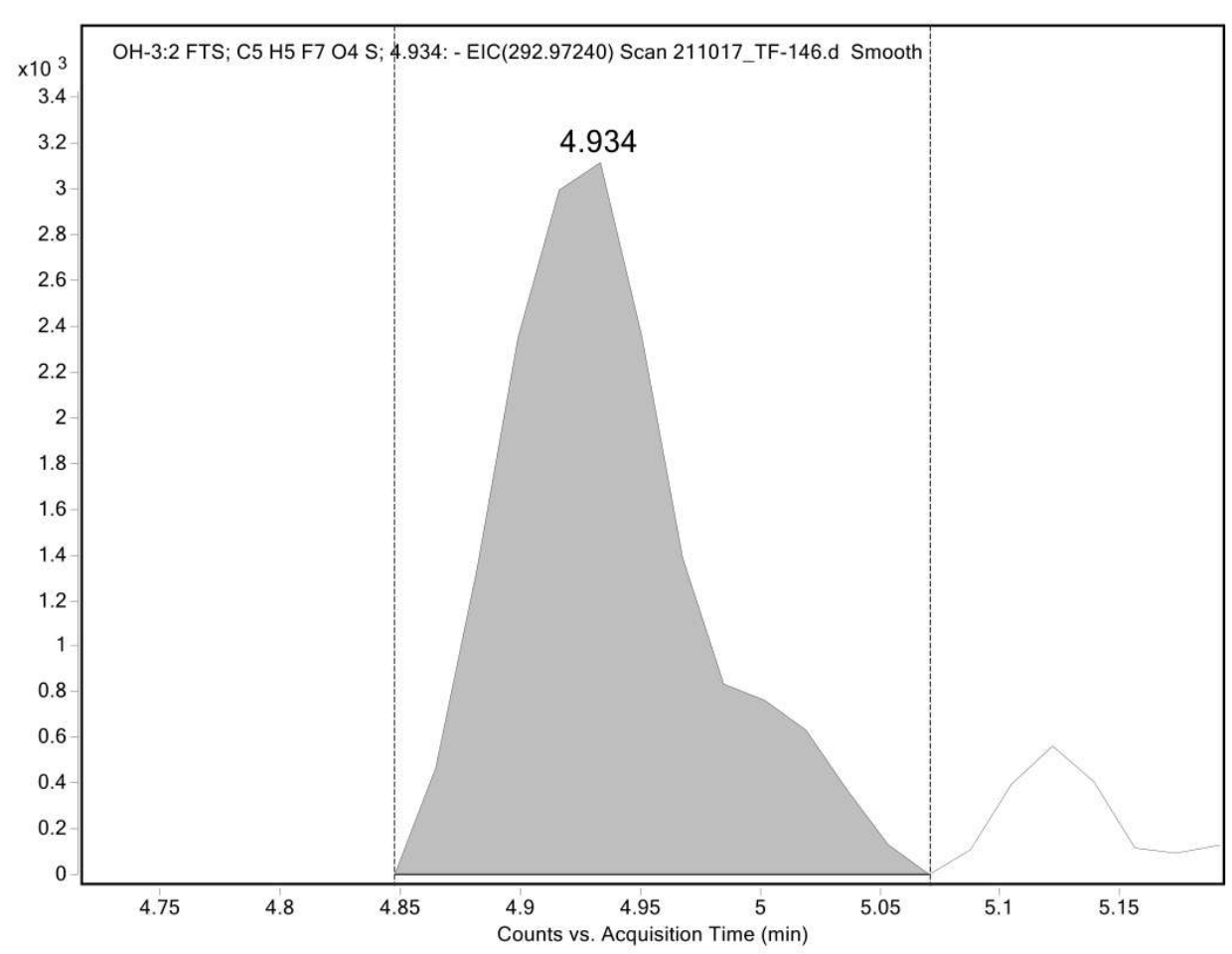

MS1

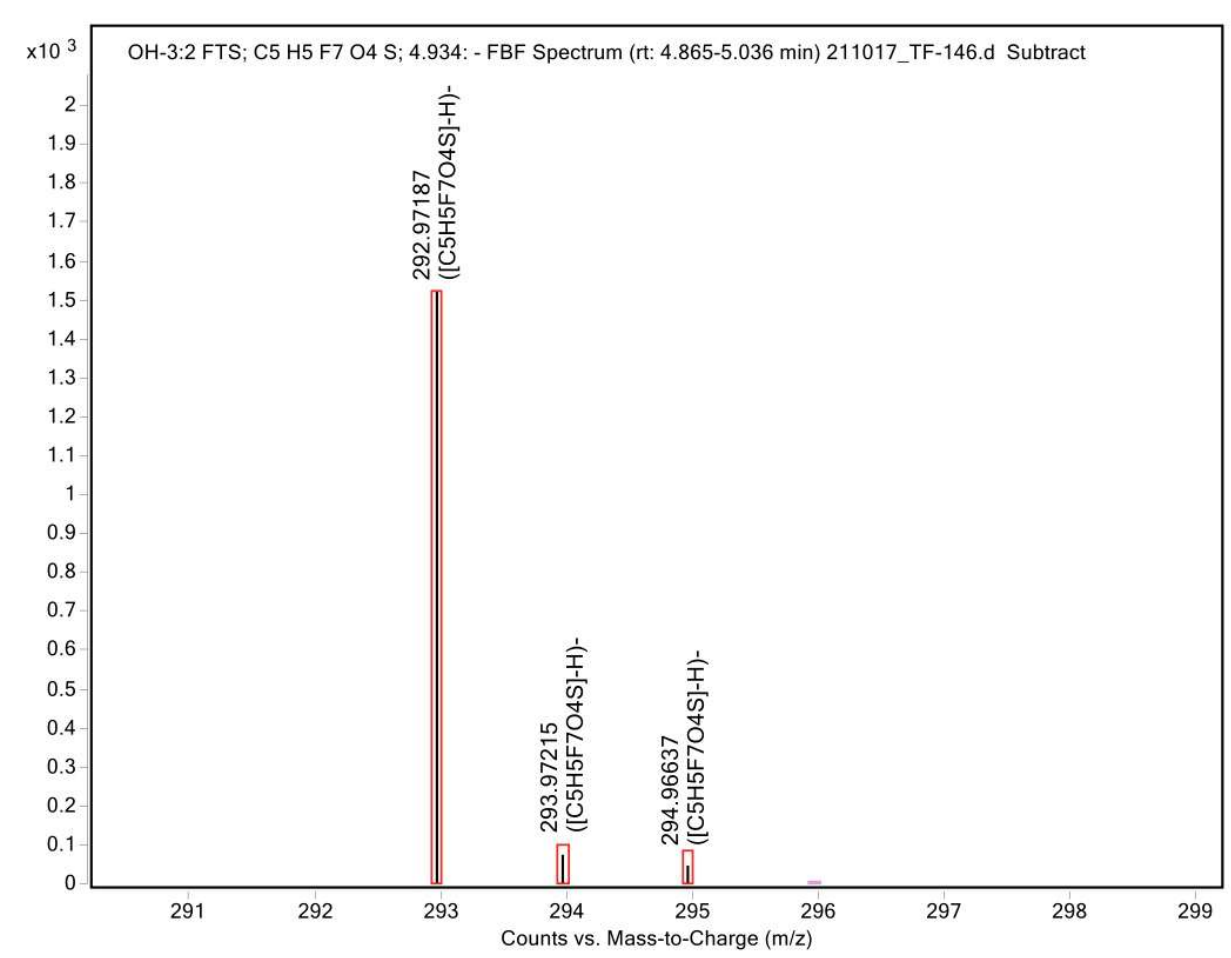

MS2 (All Ions)

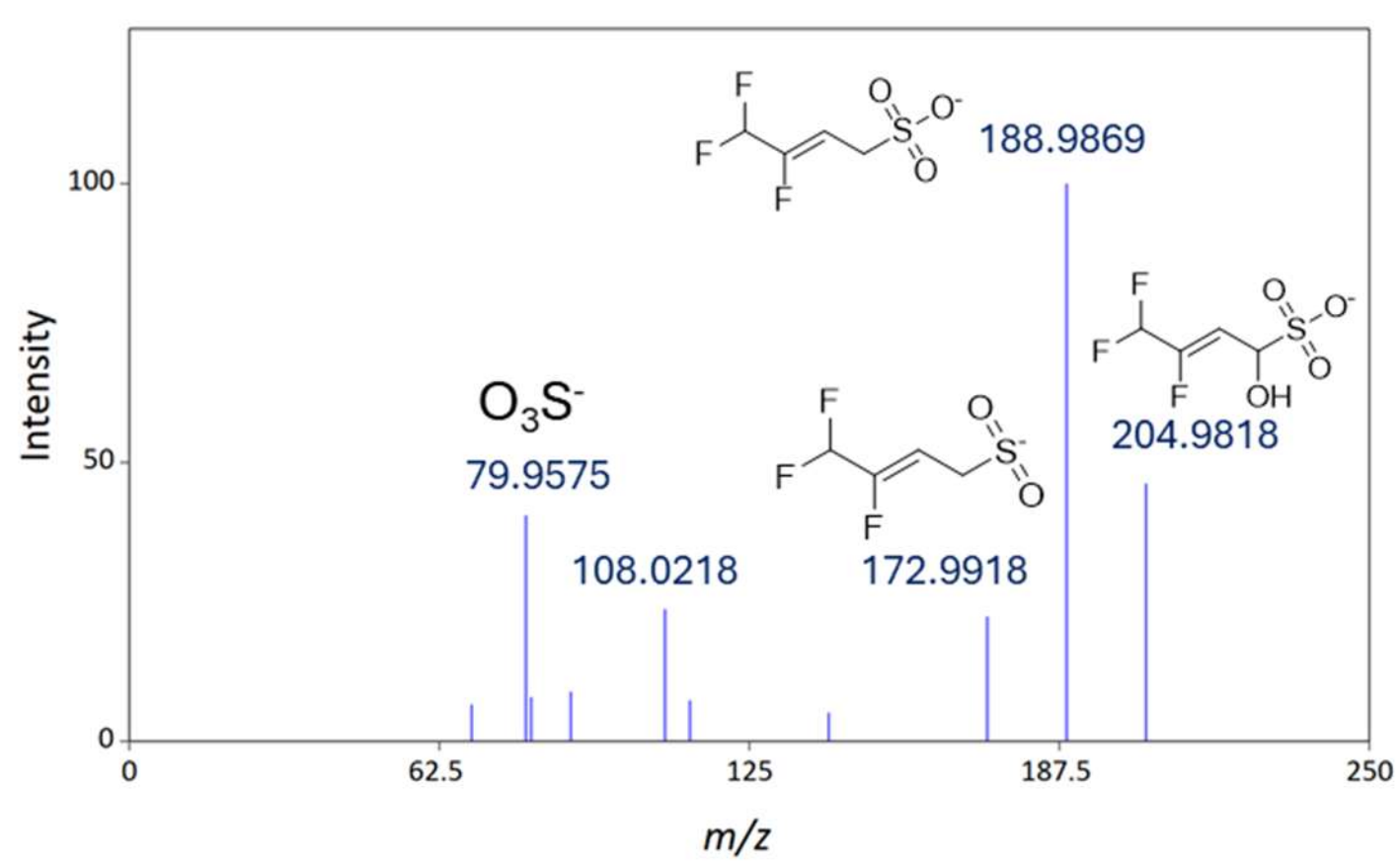

m/z	formula	mass error (ppm)
79.9574	[O3S]-	1.14
108.0228	[C3H5FO3]-	-9.74
172.9890	[C4H4F3O2S]-	16.35
188.9839	[C4H4F3O3S]-	15.89
204.9788	[C4H4F3O4S]-	14.46

8:2 FTOBzA ($C_{17}H_9F_{17}O_3$) - CL3b

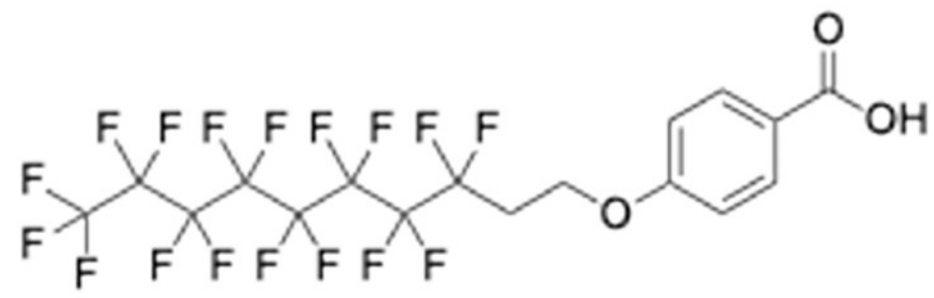

EIC

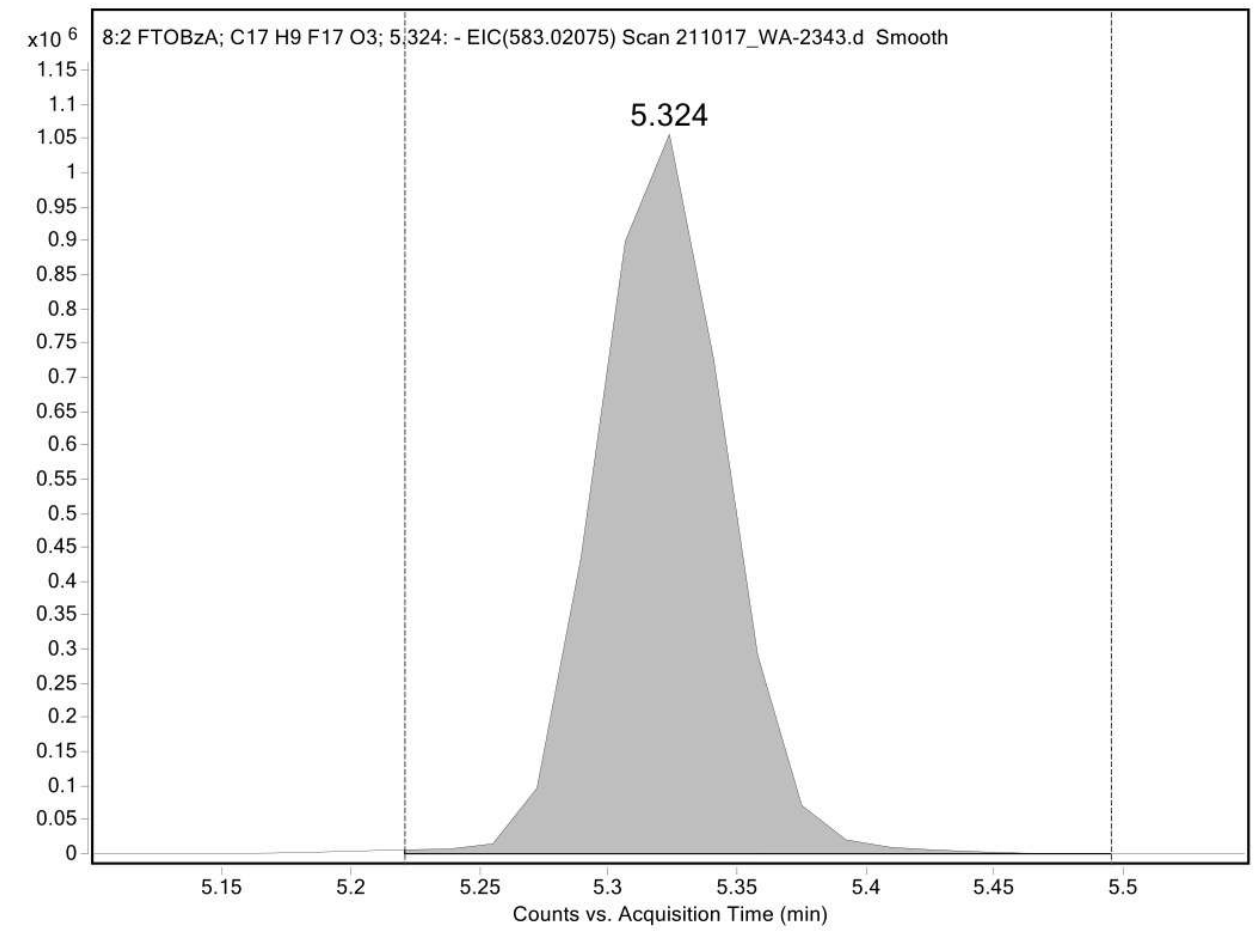

MS1

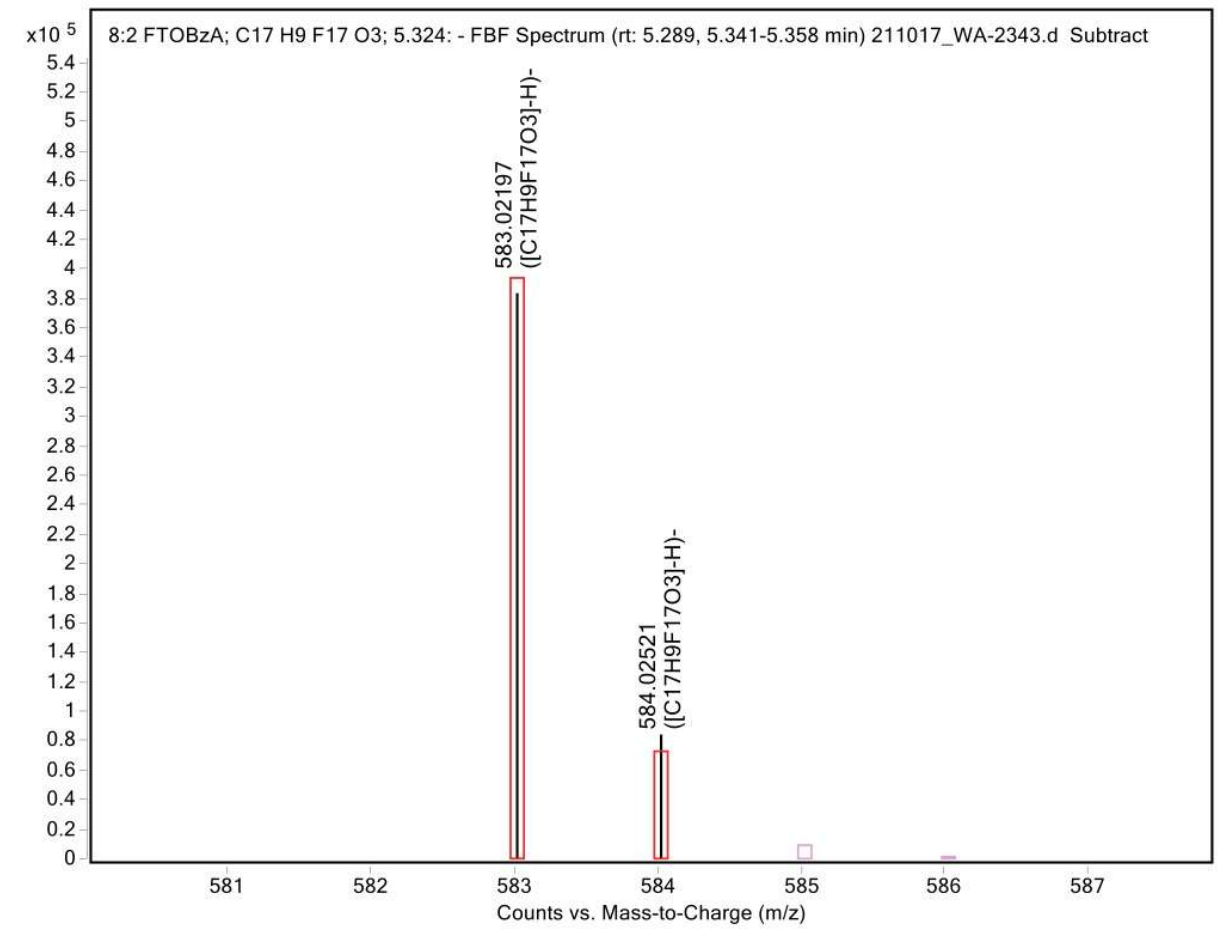

MS2 (from Data-Dependent Analysis; CE = - 20 eV)

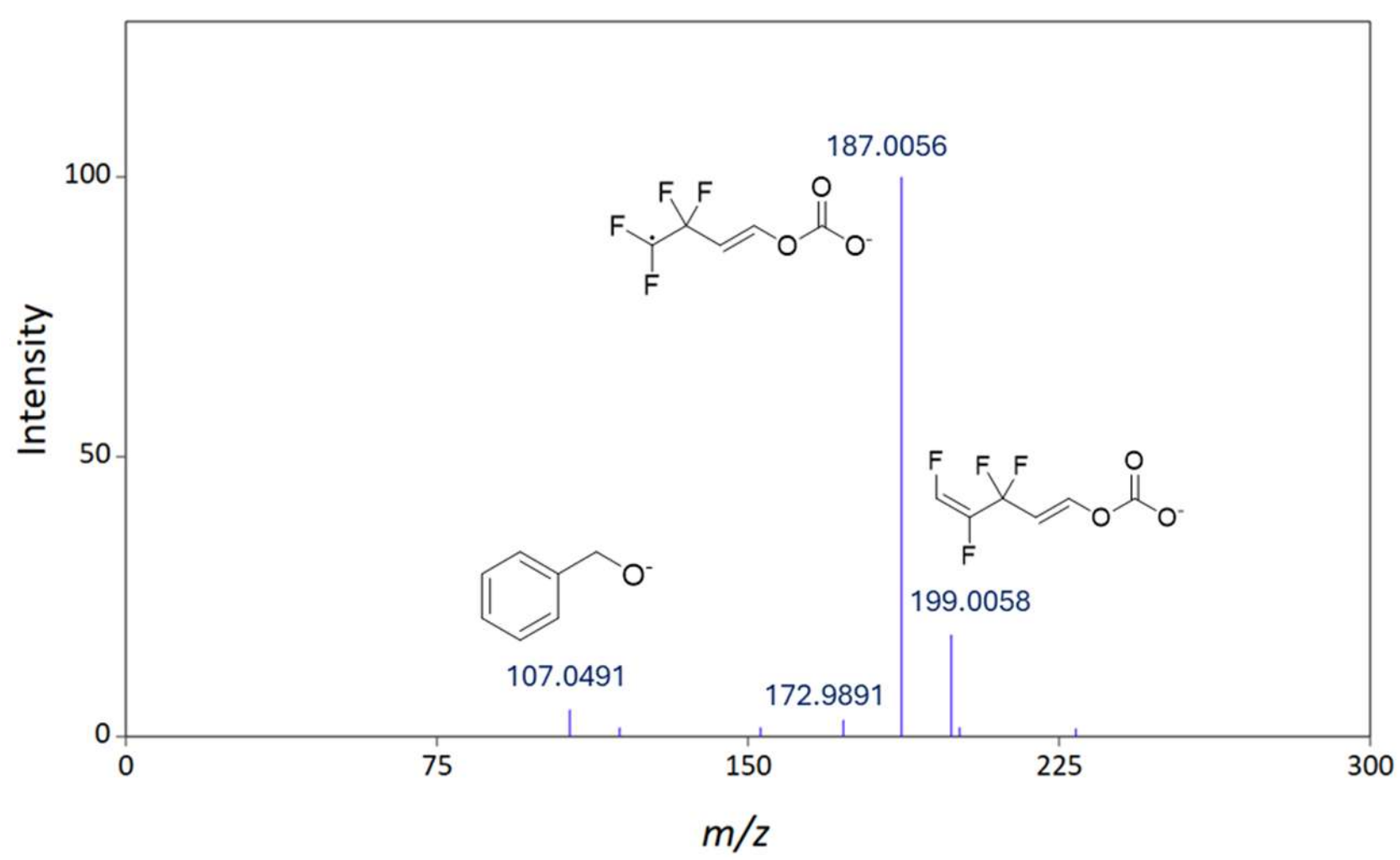

m/z	formula	mass error (ppm)
107.0502	[C7H7O]-	-10.47
172.9867	[C4HF4O3]-	13.64
187.0024	[C5H3F4O3]-	17.20
199.0024	[C6H3F4O3]-	17.32

UPFE2OA – CL3b

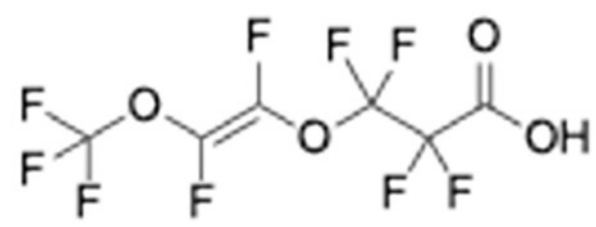

EIC

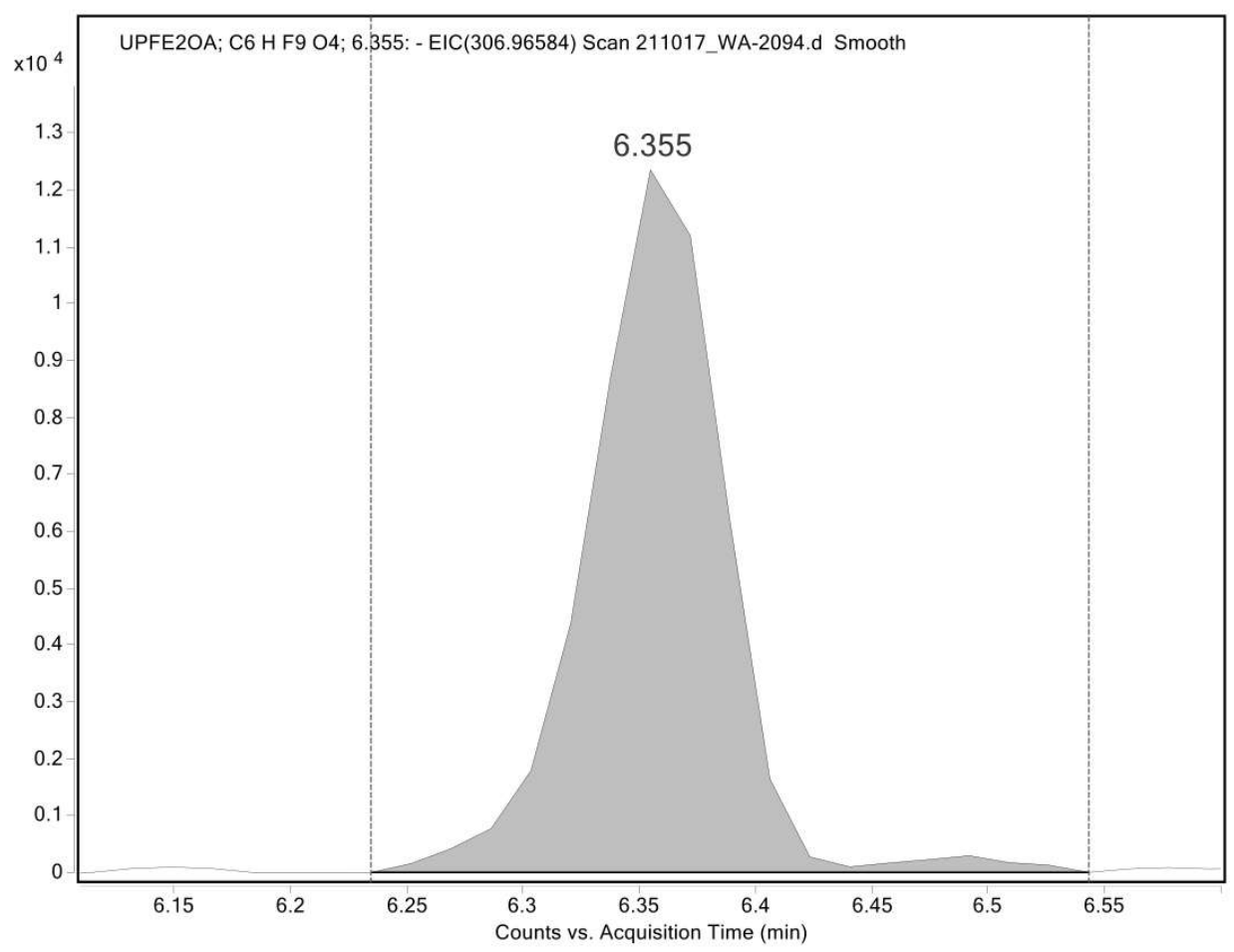

MS1

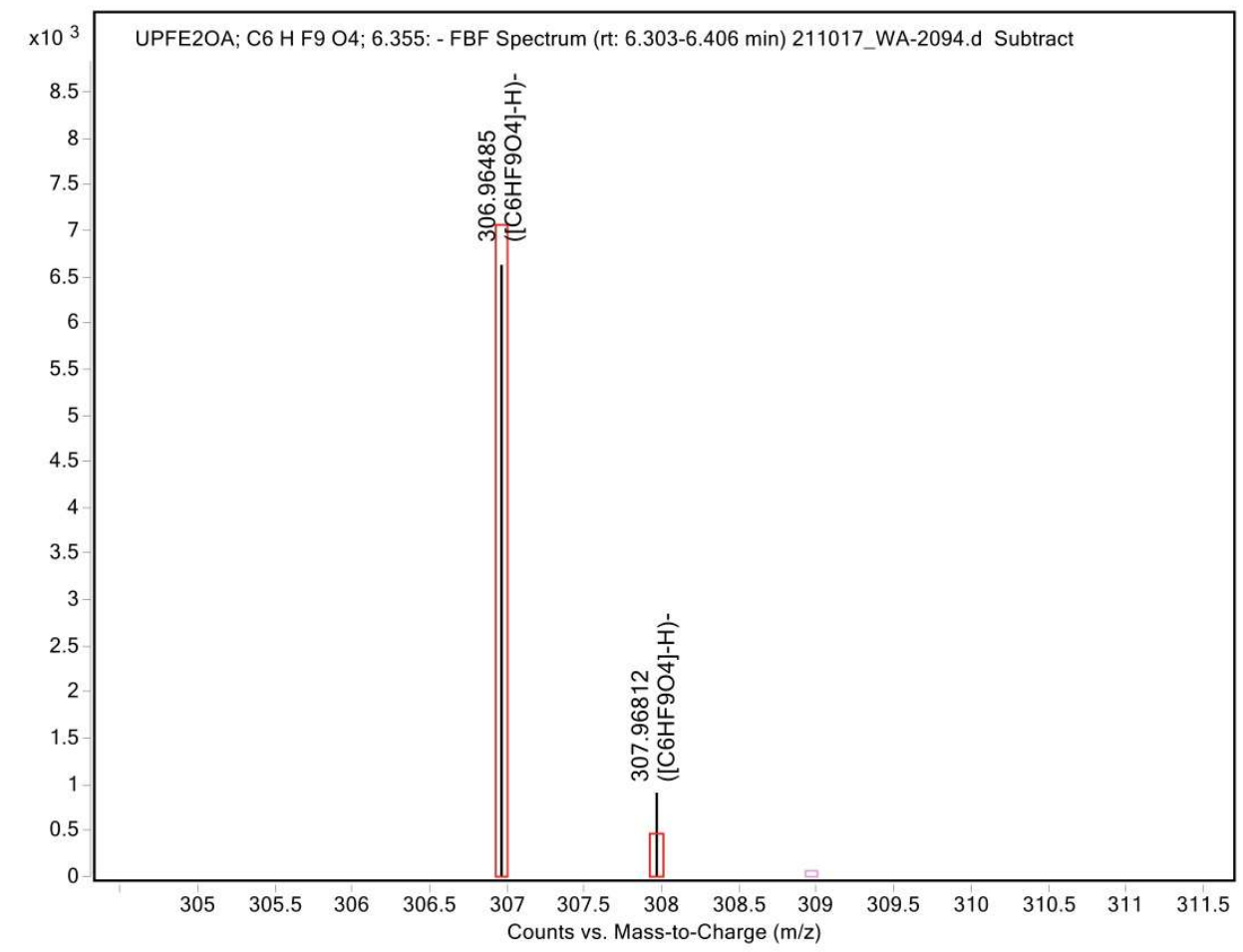

MS2 (All Ions)

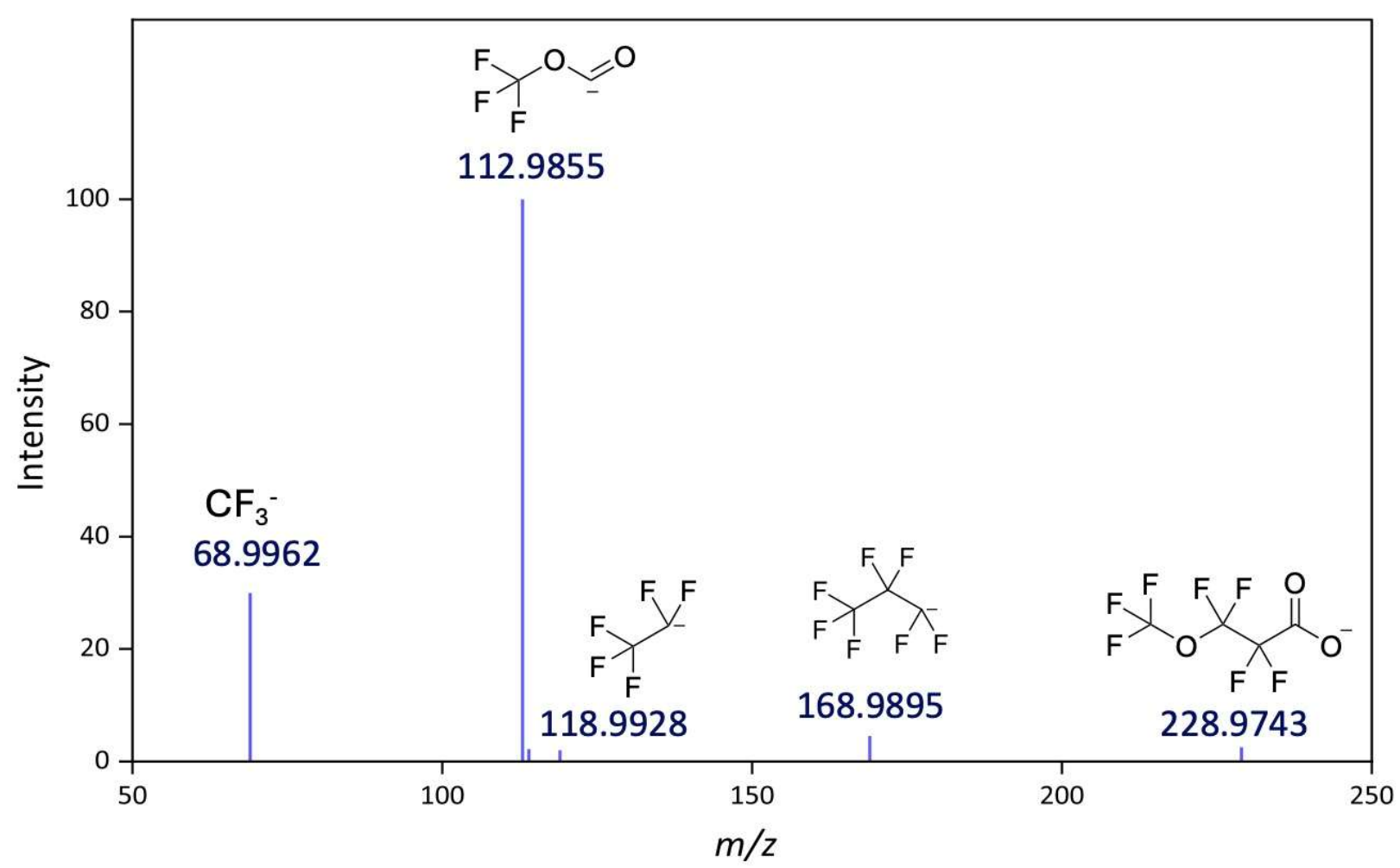

m/z	formula	mass error (ppm)
68.9958	[CF3]-	6.24
112.9856	[C2F3O2]-	-1.15
113.989	[C2F3O2]-	4.3
118.9926	[C2F5]-	1.82
168.9894	[C3F7]-	0.83
228.9741	[C4F7O3]-	1.03

H-PFOS – CL3a (positional isomer candidate)

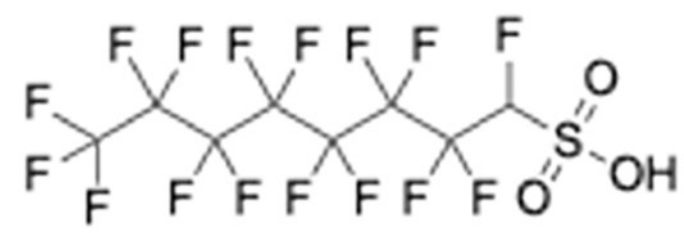

EIC

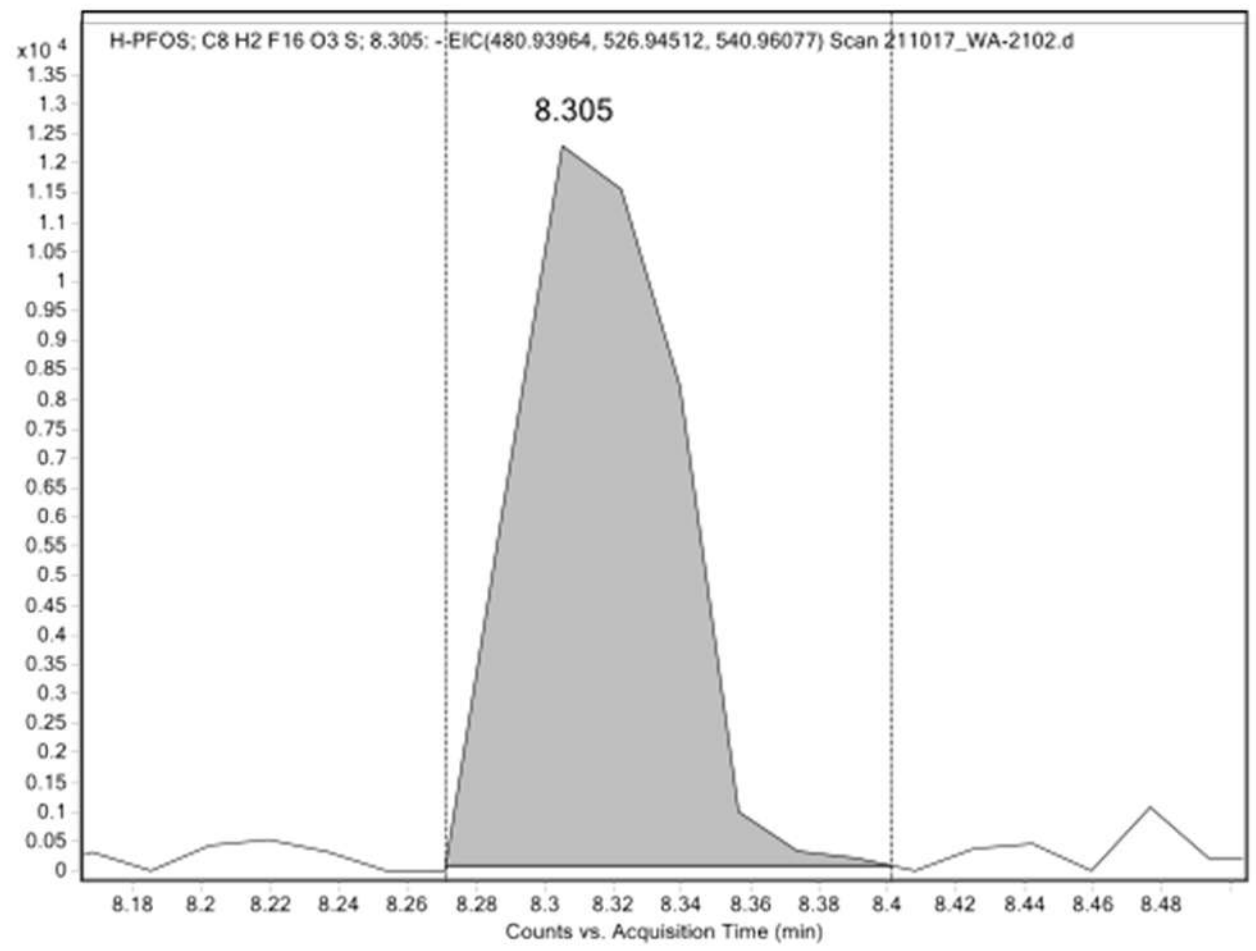

MS1

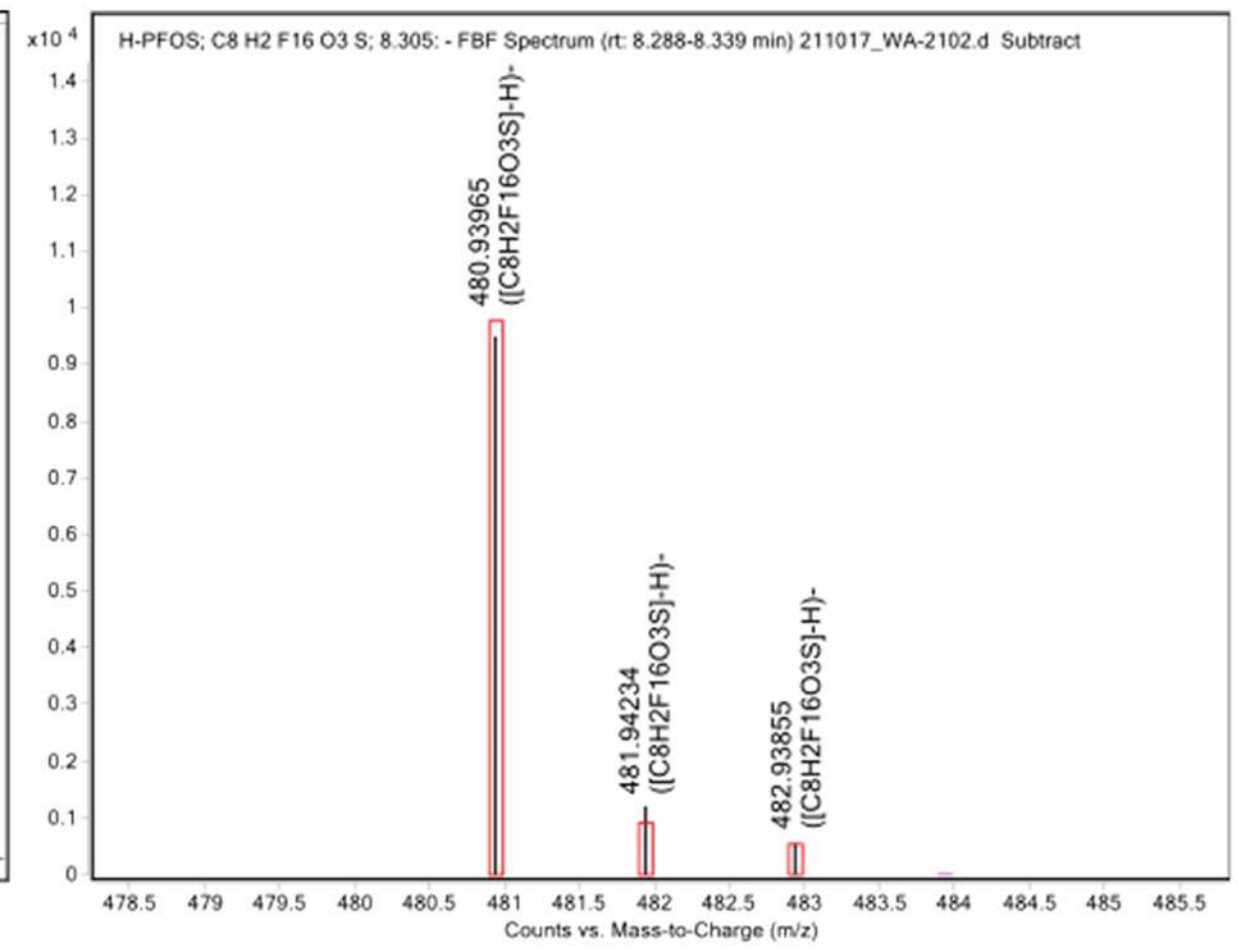

MS2 (All Ions)

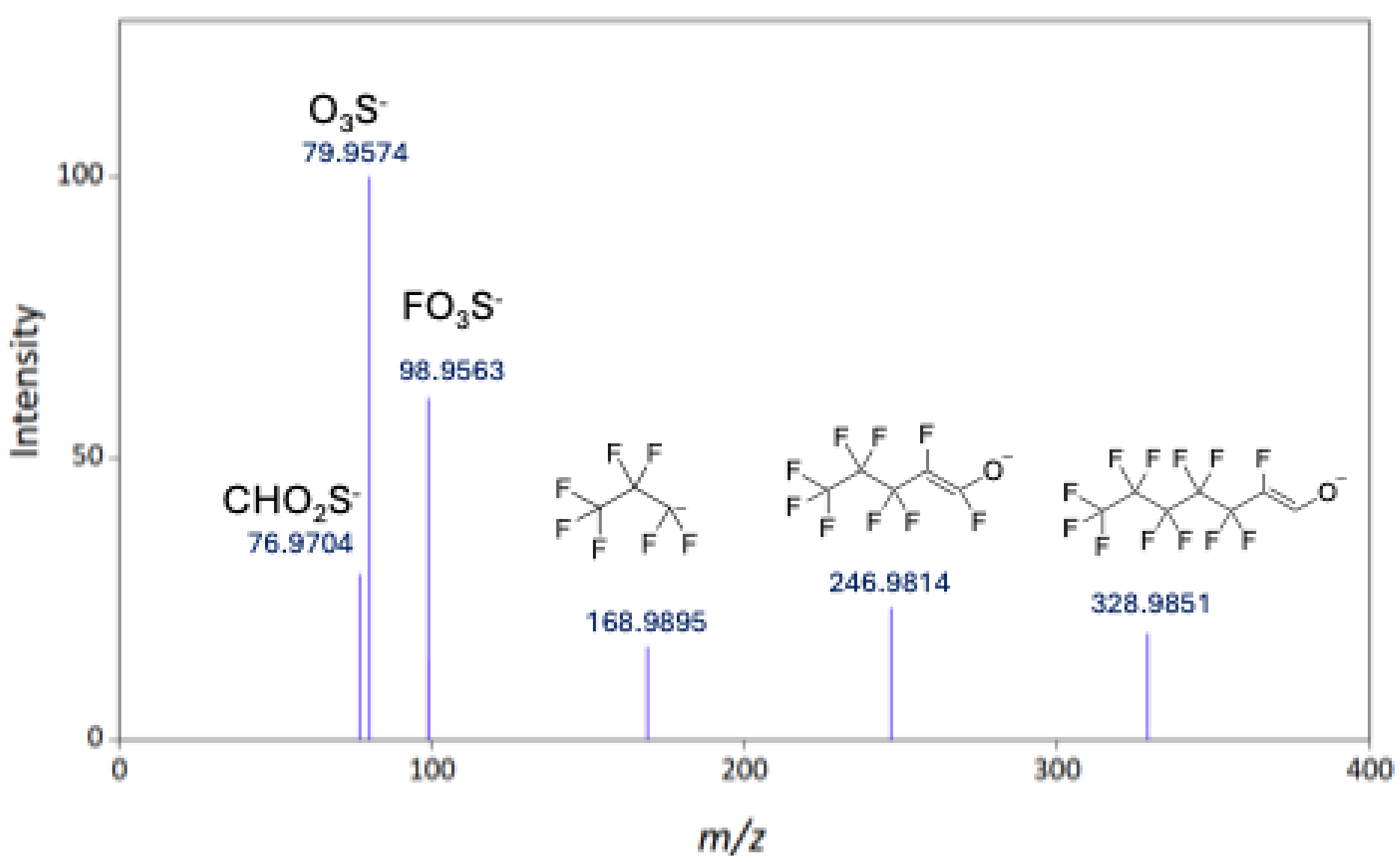

m/z	formula	mass error (ppm)
76.9703	[CHO2S]-	2.01
79.9574	[O3S]-	0.79
98.9558	[FO3S]-	5.22
168.9894	[C3F7]-	0.60
246.9811	[C5F9O]-	1.16
328.9841	[C7HF12O]-	2.94

<u>CONFIDENCE LEVEL 3d:</u> Two chlorinated perfluoroalkyl sulfonates (Cl-PFSAs) were assigned CL3d. For these identifications, only one fragment ([O3S]-) was discernible in All Ions MS/MS spectra; however, confidence was increased by their inclusion in a homologous series with Cl-PFOS (CL1b). Representative extracted ion chromatograms (EICs), MS1, and the homologous series plot are shown.

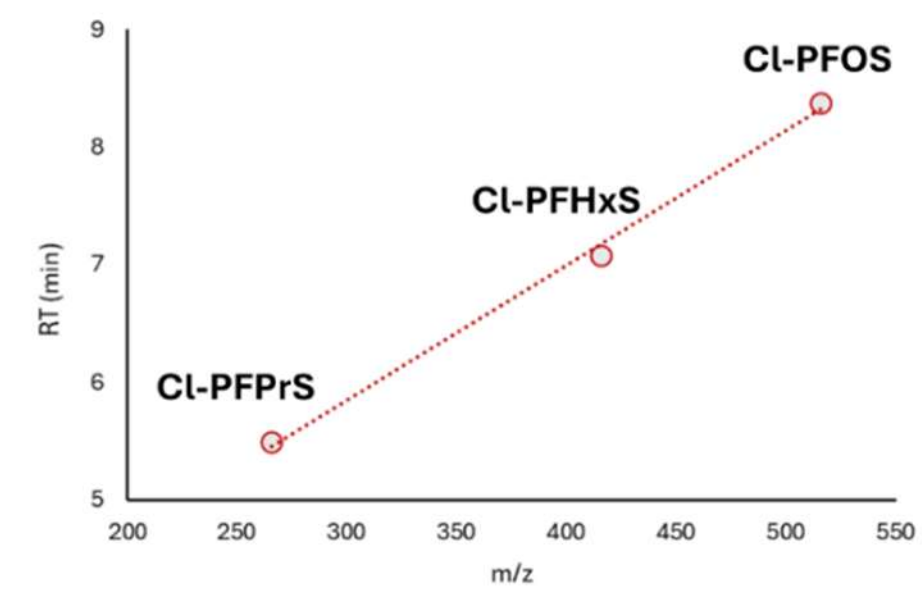

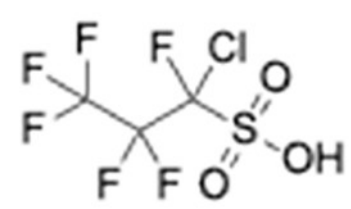

Cl-PFPrS – CL3b

EIC MS1

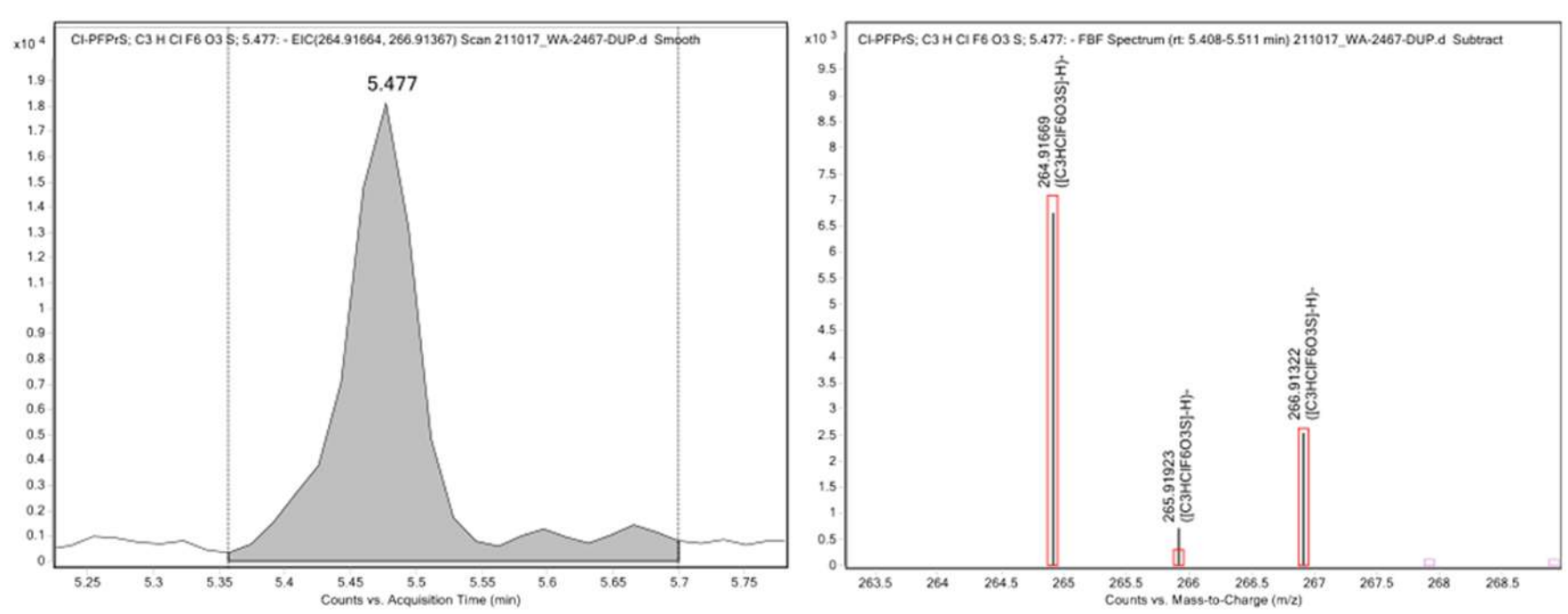

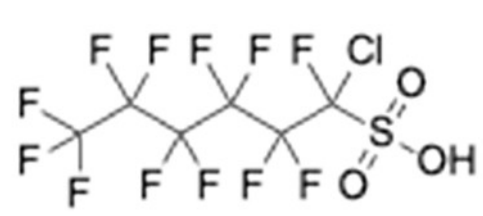

Cl-PFHxS – CL3d

EIC MS1

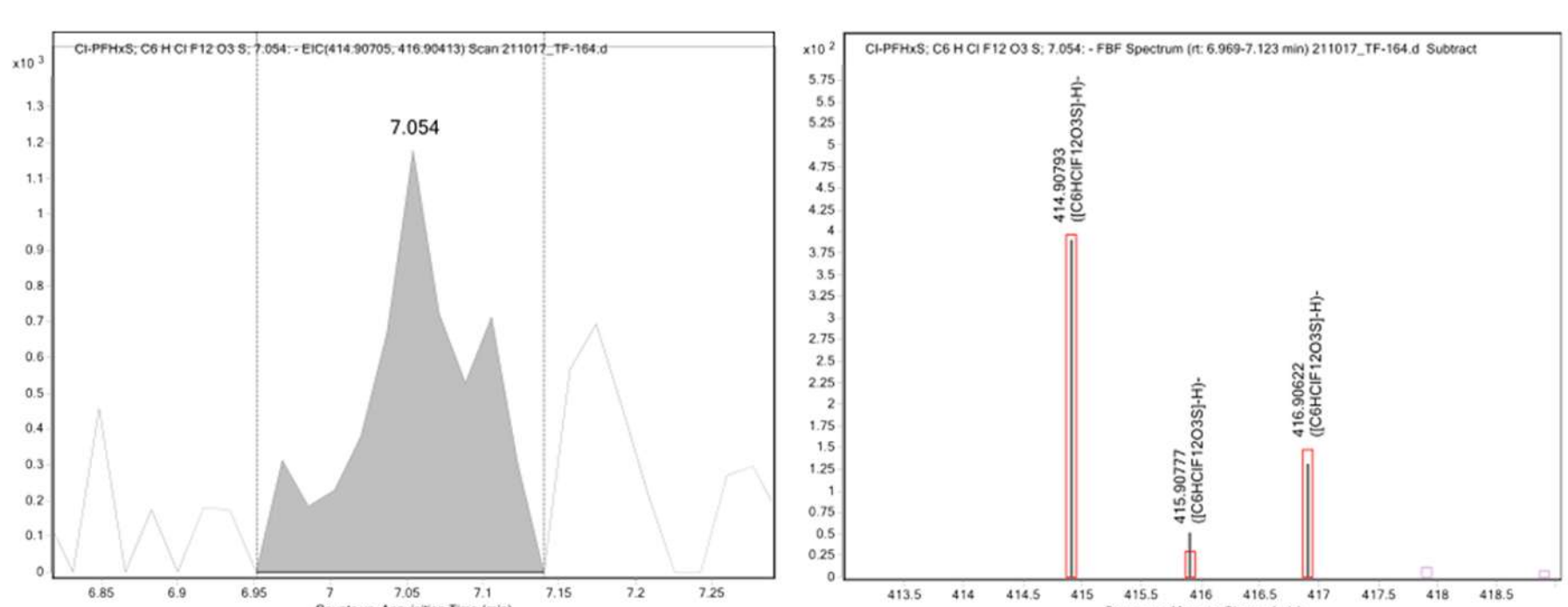

Supplement: Supplementary file 1 [file es6c05168_si_002.pdf]
